# Descriptive multi-agent epidemiology via molecular screening on Atlantic salmon farms in the northeast Pacific Ocean

**DOI:** 10.1038/s41598-020-78978-9

**Published:** 2021-02-10

**Authors:** Andrew W. Bateman, Angela D. Schulze, Karia H. Kaukinen, Amy Tabata, Gideon Mordecai, Kelsey Flynn, Arthur Bass, Emiliano Di Cicco, Kristina M. Miller

**Affiliations:** 1 Pacific Salmon Foundation, Vancouver, Canada; 2 Ecology and Evolutionary Biology, University of Toronto, Toronto, Canada; 3 Molecular Genetics, Fisheries and Oceans Canada, Nanaimo, Canada; 4 Department of Medicine, University of British Columbia, Vancouver, Canada; 5 Forest and Conservation Sciences, University of British Columbia, Vancouver, Canada

**Keywords:** Ecological epidemiology, Assay systems, Infectious-disease diagnostics, Pathogens

## Abstract

Rapid expansion of salmon aquaculture has resulted in high-density populations that host diverse infectious agents, for which surveillance and monitoring are critical to disease management. Screening can reveal infection diversity from which disease arises, differential patterns of infection in live and dead fish that are difficult to collect in wild populations, and potential risks associated with agent transmission between wild and farmed hosts. We report results from a multi-year infectious-agent screening program of farmed salmon in British Columbia, Canada, using quantitative PCR to assess presence and load of 58 infective agents (viruses, bacteria, and eukaryotes) in 2931 Atlantic salmon (*Salmo salar*). Our analysis reveals temporal trends, agent correlations within hosts, and agent-associated mortality signatures. Multiple agents, most notably *Tenacibaculum maritimum*, were elevated in dead and dying salmon. We also report detections of agents only recently shown to infect farmed salmon in BC (Atlantic salmon calicivirus, Cutthroat trout virus-2), detection in freshwater hatcheries of two marine agents (*Kudoa thyrsites* and *Tenacibaculum maritimum*), and detection in the ocean of a freshwater agent (*Flavobacterium psychrophilum*). Our results provide information for farm managers, regulators, and conservationists, and enable further work to explore patterns of multi-agent infection and farm/wild transmission risk.

## Introduction

The recent rapid pace with which marine organisms have been domesticated^1^ has elevated concern about diseases of cultured aquatic organisms^2^. As with agriculture before it^3^, aquaculture alters disease dynamics through densification of host populations and provision of novel transmission pathways, impacting both cultured and wild species^4^. As global demand for seafood continues to grow, wild-capture fishery production has plateaued^5^, and disease threatens to halt the growth of the aquaculture industry in some parts of the world^6^. Understanding infection and disease patterns in aquaculture is critical for the industry, human food production, and wild species alike.

Atlantic salmon (*Salmo salar*) aquaculture, increasing globally since the 1970s and now dwarfing wild Atlantic salmon capture^7^, has struggled with disease since its inception^8^. Several high-profile cases, among them those of parasitic sea lice, infectious pancreatic necrosis virus, and infectious salmon anemia virus, have impacted the industry and at times imperilled sympatric wild salmonids^9–11^. While government disease regulations vary among jurisdictions, the salmon-farming industry has become dominated by a few large companies that exploit economies of scale^12^, creating the potential for rapid improvements in disease management through corporate policy. Thus, lessons learned in a given salmon-farming region hold promise for other regions. Disease management in practice, however, does not always realise this potential. In particular, multiple countries have repeated the same mistakes in “managing” infectious salmon anemia (ISA)^11,13^.

Studying infectious agents in an aquaculture setting is important for multiple reasons.

First, effective disease management on salmon farms requires an understanding not only of acute disease outbreaks but also of chronic disease and sub-clinical infections involving potential disease agents. Such low-intensity infections may create production challenges, without posing an overall risk to farmed or wild population viability, but may also constitute standing populations from which more virulent strains can evolve, as in the case of infectious salmon anemia virus^14^.

Second, farm studies can yield valuable information about how infections play out within populations. Although natural mortality in wild salmon is often upwards of 90%^15^, marine predators, like many of their terrestrial counterparts^16^, preferentially select parasitized and diseased prey^17–19^. Mortality in wild fish is thus rarely observable^18^ as dying fish either drop out of the water column or are quickly consumed by predators. Open-net salmon farming, allowing free-flow of water with the surrounding marine environment, offers the potential to understand disease progression and associated mortality in a semi-natural setting but with an almost complete absence of predators.

Third, farm studies promise insight into infectious agent exchange between wild and farmed fish. The interface between wildlife and livestock presents a nexus for shared^20,21^ and emergent^22^ infectious disease. In a wildlife/livestock disease context, surveillance and monitoring for disease-agent presence, prevalence, and infection intensity are critical for disease management in both wildlife and livestock^22,23^. Relevant considerations include the speed with which farmed fish become infected, infectious agents’ presence as acute or chronic infections, and correspondence between infectious agents and seasonal patterns of wild-fish migration.

While captive populations offer opportunities for improved understanding of infection and disease, little published information describes the infection status of farm populations outside of mortality events or details differential patterns of infection between live and moribund fish. We note that corporate monitoring data and targeted longitudinal studies have previously been used to understand health and disease development^24,25^, but common single-agent focus has limited understanding of the role of co-infection in disease development, and a general focus on sick and dying fish has limited our understanding of infection progress and resulting pathogen release. Moreover, if we are concerned about biosecurity and the risk of pathogen exchange among farms and between farmed and wild salmon, infection levels in the population as a whole, rather than solely in dying fish, will provide a more accurate assessment of transmission risk.

We report findings from a multi-year, multi-infective-agent monitoring program^26^, focused on farmed Atlantic salmon in Pacific Canada. This work forms part of a broader research effort using next-generation sequencing in pathogen discovery, followed by high-throughput genetic screening techniques, (developed in parallel and first reported for the human-health field^27^) to monitor infective agents in multiple salmon populations^18,26^. Here, we focus on results from a research-directed screening program, conducted at regular intervals on four Atlantic salmon farms throughout their production cycles. We characterise time series for dozens of infective agents, identify agents associated with mortality, and provide information for future epidemiological study.

## Materials and methods

**Figure 1:**
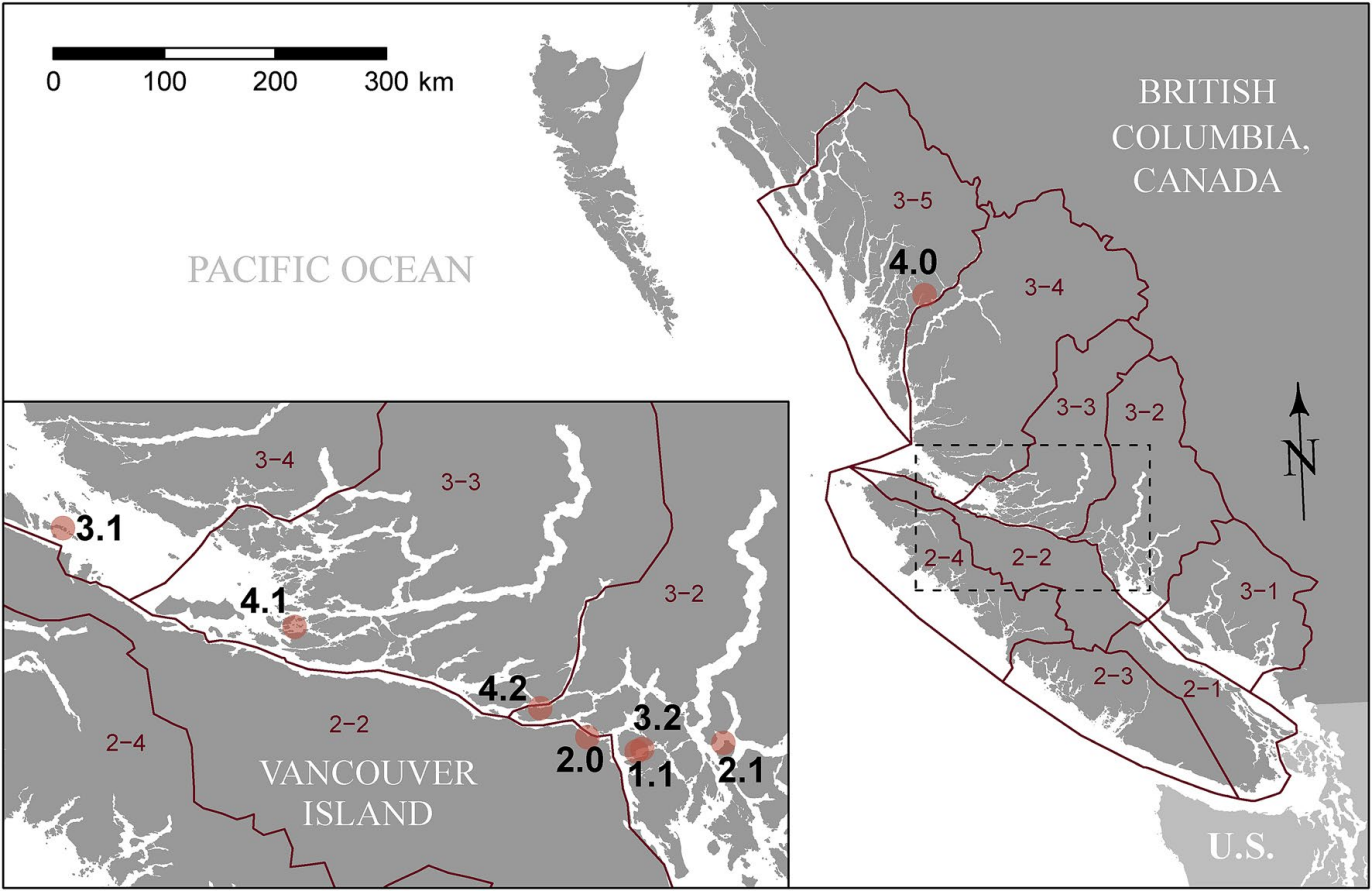
Atlantic salmon-farm locations (points) for the four cohorts from which samples were collected for this study, in relation to Fisheries and Oceans Canada’s Aquaculture Management Zones (2–1 through 3–5). For each farm location, labelled *x*.*y*, *x* indicates a cohort of salmon (1 through 4) while *y* indicates successive locations of that cohort: 0—freshwater hatchery, 1 & 2 (in some cases)—sequential saltwater net-pen locations.

Four cohorts of farmed Atlantic salmon (in BC’s Fish Health Zones A3.2, A3.3, and A3.4; Fig. 1, Table 1) were sampled repeatedly throughout their marine production cycles, from ocean entry to the onset of harvest. Both live individuals, after euthanisation, and recently dead or dying individuals were sampled. Tissue from each sampled fish was genetically assayed for 47 different infectious agents (including viruses, bacteria, and metazoans; Table 2). After field sampling and genetic workup, we fit descriptive statistical models to genetic prevalence and intensity time series for individual agents. We modelled temporal trends, calculated agent correlations and overall infection burdens, and looked for differences between live and dead/dying fish.

**Table 1:** Movement history for farmed Atlantic salmon used in this study. Geographic locations of individual facilities are shown in Fig. 1.

cohort	Facility type	facility code (Fig. 1)	DFO Fish Health Zone	Entry (initial)	Exit (final)
1	Farm	1.1	3–2	Apr 2013	Mar 2015
2	Hatchery	2.0	2–2	-	Nov 2013
	Farm	2.1	3–2	Nov 2013	Sep 2015
3	Farm	3.1	3–4	Apr 2013	Dec 2013
	Farm	3.2	3–2	Nov 2013	Apr 2015
4	Hatchery	4.0	3–5	-	Oct 2013
	Farm	4.1	3–3	Oct 2013	Aug 2014
	Farm	4.2	3–2	Aug 2014	Sep 2015

**Table 2:** Low-prevalence infective agents in initial rounds of mixed-tissue sample screening in farmed Atlantic salmon.

Organism	Organism type	Test code	Status	Freshwater		Saltwater	
				Tests‡	Prevalence	Tests‡	Prevalence
Infectious pancreatic necrosis virus	Virus	IPNV	Known	0	–	933	0
Infectious salmon anemia virus	Virus	ISAV-7	Known	0	–	933	0
Infectious salmon anemia virus	Virus	ISAV-8	Known	0	–	933	0
*Oncorhynchus masou* herpes virus	Virus	OMV	Known	0	–	933	0
Piscine myocarditis virus	Virus	PMCV	Known	0	–	933	0
Salmon alphavirus	Virus	SAV	Known	0	–	933	0
*Aeromonas hydrophila*	Bacterium	Ae_hyd	Known	0	–	933	0
*Moritella viscosa*	Bacterium	Mo_vis	Known	0	–	933	0.0096
*Spironucleus salmonicida*	Flagellate	Sp_sal	Known	0	–	933	0
*Gyrodactylus salaris*	Fluke	Gy_sal	Known	0	–	933	0
*Nucleospora salmonis*	Microsporidian	Nu_sal	Known	0	–	931	0.0150

^‡^successful tests only (i.e. those without evidence of control cross contamination, poor amplification curves, or low housekeeping gene signals).

### Ethics statement

All work with animals was performed according to the Canadian Council on Animal Care’s (CCAC) Guide to the Care and Use of Experimental Animals, and project protocols were approved by the federal department of Fisheries and Oceans Canada (DFO) through its Pacific Region Animal Care Committee (Animal Use Protocol Number: 13-008). Live-sampled fish were euthanised via overdose of tricaine methanesulfonate (Syndel laboratories Ltd., Nanaimo BC, Canada). All tissue samples involved were collected under a Material Transfer Agreement between the BC Salmon Farmers Association and Fisheries and Oceans Canada.

### Data series

#### Infectious-agent data

##### Farm schedule and sample collection

Of the four farm cohorts in the study, two entered the ocean in the spring, and another two entered in the fall (Table 1). Each of the two salmon-farming companies involved, Cermaq and Marine Harvest (recently renamed MOWI) Canada, granted access to one spring-entry and one fall-entry cohort. Two of the cohorts were transferred between farm locations approximately halfway through their respective production cycles (Table 1).

For each cohort, live fish were sampled at an approximate rate of 30 individuals every two weeks for the first five months, and 20 individuals per month thereafter until harvest. At each sampling visit, up to 20 dead or dying (moribund) fish from across the farm site were also sampled. For each of the two fall-entry cohorts, fish were also sampled in the respective hatcheries, with approximately 100 juvenile fish sampled prior to vaccination, and approximately 100 sampled post vaccination. Company veterinarians selected a single net pen per site visit from which to sample live fish, and rotated their selections to reduce handling stress among pens. Industry constraints meant that dead and dying fish from certain mortality or disease events were unavailable, in some cases resulting in reduced dead/dying samples in months with elevated mortality.

Each sampled fish was euthanised if living and then dissected for gill, liver, kidney, heart, and brain. Dissections were conducted with separate external and internal tools (2 full sets) for each fish, and tools were not re-used during the sampling events. Following their use, tools were treated to a regime of water, bleach, water, ethanol, and flame. Fish were dissected using aseptic technique one fish at a time into individual tubes or RNAlater that were closed and sealed once tissue was placed inside. Sampled tissues were held at 4 °C overnight then stored at - 80 °C until laboratory analysis. After each individual fish was dissected, the operating theatre was completely broken down and waste removed from the dissection area. The surfaces were wiped with bleach and then 70% ethanol and a new dissection theatre assembled with fresh gloves, new outside and inside tools, and tubes. Full dissection kits were assembled for each farm site, and following each site visit the entire kit was sterilized.

#### Laboratory analysis

We used a Fluidigm BioMark™ HD microfluidics-based qPCR platform, developed and validated for salmon infective-agent monitoring^18,26^, to screen samples for viral, bacterial, and eukaryotic agents known to infect and potentially cause disease in salmon worldwide. The BioMark™ platform employs assays designed to assess presence and concentration of specific targeted nucleic acid sequences, and is sensitive to between one and three sequence copies per test volume^26^. The data we report are estimates of the number of target gene copies per μL of nucleic-acid solution, extracted from mixed-tissue homogenates and standardised to a fixed total nucleic-acid concentration (see Supplemental Information). We did not attempt to relate gene copies directly to infective-agent numbers, as such work was outside the scope of our study.

Substantial measures were taken to maximise data quality. We used a house-keeping gene assay to gauge sample quality and to ensure that nucleic acid degradation had not occurred. Each assay for an infectious agent was run in duplicate for each sample. Analytical sensitivity and specificity, and assay repeatability of the BioMark™ platform have been previously evaluated^26^. Extraction (DNA and RNA) and analysis protocols are described elsewhere^26,28^ and we provide details in the Supplementary Information. Critically, we ran a series of negative controls to minimise false-positive results: negative extraction controls on RNA extraction plates, negative cDNA controls and no RT controls for the cDNA step, negative STA and no primer-STA controls for the specific target amplification step, and a blank buffer at the time of chip loading. We did not apply a limit-of-detection cutoff to the data we analysed, but we did discard results for which duplicate tests run on the same sample disagreed with respect to presence of an agent. We considered successful tests to be those without evidence of control cross contamination, poor amplification curves, or low housekeeping gene signals, and we discarded results from unsuccessful tests, while retaining successful results for other tests run on the same sample, as appropriate.

Laboratory screening occurred in two main phases. Initially, we screened 933 mixed-tissue samples for a suite of 47 agents (status “known” in Tables 2 and 3). Some of the corresponding results have been reported elsewhere^29^. In subsequent screening, we omitted eleven of the initially known agents (Table 2), which displayed very low prevalence or were not detected at all. In their place during the second phase of screening, we tested for eleven additional viruses, which had not been discovered at the time of the initial experimental design^30,31^ (status “new” in Table 3). Of these agents, two—Atlantic Salmon Calicivirus (ASCV) and a recently sequenced variant of Cutthroat trout virus (CTV-2)—displayed high prevalence. To fill in the time series and enable analysis, we re-assayed 215 mixed-tissue samples for ASCV and CTV-2, and assayed a further 16 samples that had not previously had mixed tissues successfully assayed (231 fill-in samples in total), using the Applied Biosystems 7900HT platform. We chose these fill-in samples to maximise temporal representation across farms, and we did not consider their status with respect to other agents during selection.

**Table 3:** Infective agents in mixed-tissue samples of farmed Atlantic salmon.

Organism	Organism type	Test code	Status	Freshwater		Saltwater	
				Tests‡	Prevalence	Tests‡	Prevalence
Salmon pescarena-virus-1†	Virus	SPAV-1	New	427	0	1611	0
Salmon pescarena-virus-2†	Virus	SPAV-2	New	425	0	1605	0.0019
Atlantic salmon calicivirus	Virus	ASCV	New	423	0.1300	1835	0.5384
Pacific salmon nidovirus†	Virus	PSNV	New	427	0.0023	1611	0.0006
Cutthroat trout virus-2†	Virus	CTV-2	New	426	0.2840	1808	0.6610
Putative Narna-like virus†	Virus	NARNAV	New	427	0	1606	0.0212
Orthomyxovirus†	Virus	ORTHO	New	427	0	1611	0.0006
Chinook aquareovirus†	Virus	CAV	New	426	0.0023	1611	0.0012
Poxvirus	Virus	SGPX	New	427	0	1611	0
Putative RNA virus†	Virus	RNAV	New	427	0	1611	0.0006
Putative totivirus†	Virus	TOTIV	New	427	0.0023	1607	0.0137
Erythrocytic necrosis virus	Virus	ENV	Known	427	0	2470	0.0049
Infectious hematopoietic necrosis virus	Virus	IHNV	Known	427	0	2471	0.0004
Piscine orthoreovirus	Virus	PRV	Known	421	0.3278	2404	0.5420
Pacific salmon parvovirus	Virus	PSPV	Known	327	0	2101	0
Viral encephalopathy & retinopathy	Virus	VERV	Known	427	0	2471	0.0012
Viral hemorrhagic septicemia virus	Virus	VHSV	Known	427	0	2471	0
*Aeromonas salmonicida*	Bacterium	Ae_sal	Known	425	0.0094	2466	0.0020
*Candidatus* Branchiomonas cysticola	Bacterium	C_B_cys	Known	426	0.3873	2464	0.0369
*Flavobacterium psychrophilum*	Bacterium	Fl_psy	Known	414	0.3382	2459	0.0130
*Piscichlamydia salmonis*	Bacterium	Pch_sal	Known	427	0	2471	0
*Piscirickettsia salmonis*	Bacterium	Pisck_sal	Known	427	0.0023	2468	0.0073
*Renibacterium salmoninarum*	Bacterium	Re_sal	Known	427	0	2472	0.0024
Strawberry disease (Rickettsia-like)	Bacterium	RLO	Known	427	0	2472	0
*Candidatus* Syngnamydia salmonis	Bacterium	C_S_sal	Known	427	0.0047	2461	0.0561
*Tenacibaculum maritimum*	Bacterium	Te_mar	Known	426	0.0423	2335	0.6081
*Vibrio anguillarum*	Bacterium	Vi_ang	Known	423	0.0331	2477	0.0153
*Vibrio salmonicida*	Bacterium	Vi_sal	Known	425	0.0965	2458	0.0260
*Yersinia ruckeri*	Bacterium	Ye_ruc	Known	427	0.0187	2468	0
*Neoparamoeba perurans*	Amoeba	Ne_per	Known	427	0	2472	0.0004
*Nanophyetus salmincola*	Fluke	Na_sal	Known	427	0	2472	0
*Sphaerothecum destruens*	Mesomycetozoan	Sp_des	Known	427	0	2471	0.0004
*Facilispora margolisi*	Microsporidian	Fa_mar	Known	427	0	2460	0.0293
*Loma salmonae*	Microsporidian	Lo_sal	Known	427	0	2472	0
*Paranucleospora theridion*	Microsporidian	Pa_ther	Known	410	0.2317	2383	0.9123
*Ceratomyxa shasta*	Myxozoan	Ce_sha	Known	427	0	2472	0
*Kudoa thyrsites*	Myxozoan	Ku_thy	Known	424	0.0755	2386	0.4438
*Myxobolus arcticus*	Myxozoan	My_arc	Known	427	0	2471	0.0020
*Myxobolus insidiosus*	Myxozoan	My_ins	Known	427	0	2472	0
*Parvicapsula kabatai*	Myxozoan	Pa_kab	Known	427	0.0023	2461	0.1187
*Parvicapsula minibicornis*	Myxozoan	Pa_min	Known	427	0	2469	0.0004
*Parvicapsula pseudobranchicola*	Myxozoan	Pa_pse	Known	427	0	2312	0.3166
*Tetracapsuloides bryosalmonae*	Myxozoan	Te_bry	Known	427	0.0070	2472	0
*Cryptobia salmositica*	Protozoan	Cr_sal	Known	427	0	2472	0
*Dermocystidium salmonis*	Protozoan	De_sal	Known	427	0	2472	0
*Ichthyophonus hoferi*	Protozoan	Ic_hof	Known	427	0	2471	0.0024
*Ichthyophthirius multifiliis*	Protozoan	Ic_mul	Known	384	0.0052	2290	0.0157

†Indicates agents newly discovered or not known from salmon in BC^30,31^.

^‡^successful tests only (i.e. those without evidence of control cross contamination, poor amplification curves, or low housekeeping gene signals).

### Statistical analyses

We analysed agent data to assess how apparent infection patterns changed over the course of a cohort’s grow-out period, in relation to season, and between live and dead fish. We further analysed multi-agent infectious burden and how agent levels were correlated across hosts.

We note that agent data exhibit sampling bias, because disproportionately few samples were available from high-mortality periods due to farm constraints, and we did not known if, or how often, live fish were sampled from pens with elevated mortality. As a result, we do not attempt to relate infectious-agent levels to on-farm mortality levels.

#### Single-agent time series

To describe patterns in disease-agent time series from the four focal Atlantic-salmon farm cohorts, we fit generalised additive models^32^ to single-agent data. GAMs provide flexible functional forms able to capture epidemiological patterns akin to those described by susceptible-infected-recovered (SIR) models^33^, although they do not share a mechanistic basis.

For each agent with sufficient detections, we fit models to prevalence and intensity responses. We define agent prevalence as the proportion of successful tests with a positive detection, and we define agent intensity as the number of gene copies (the “load”) in a sample with a positive detection. First, we fit models of agent prevalence to detection/non-detection data, assuming a binomial response and logit (log-odds) link function. Second, for the samples in which we detected a focal agent, we fit models of average infection intensity to log-transformed copy-number data, assuming a normal response and linear link function. We incorporated four predictor components in our models: (1) a smooth function of the number of days since ocean entry, parameterised with a cubic-spline basis and allowed to differ among cohorts; (2) an additional smooth function of the number of days since ocean entry to account for differences between live and dead/dying hosts, again parameterised with a cubic-spline basis and allowed to differ among cohorts; (3) a scalar effect for each cohort; and (4) a smooth term with a cyclic cubic-spline basis to account for consistent seasonal patterns across cohorts. In this way, models could capture nonlinear trends over time, allowing for different patterns between live and dead/dying fish, and among farm cohorts. For the prevalence models, we used ten knots in the smooth terms for components 1) and 2), and four knots in the smooth terms for component 4). For the intensity models, we included identical model components, except that we reduced the number of knots in 1) and 2) to eight, aiming to avoid overly flexible models for use with reduced sample sizes after omitting non-detections.

While our models were unable to reveal general patterns of infection for any agent in BC salmon farms, due to the limited number of cohorts observed, our models do allow for evaluation of temporal trends within each cohort and for comparison among groups (cohorts, live and dead/dying fish, etc.). This approach was useful to distinguish the average “signal” from the “noise” in each cohort over time, in the context of many screening tests across a large number of samples exhibiting a large range of apparent infection intensity.

In initial agent-prevalence model fitting, GAM fits in regions of the data with all-zero or all-one responses yielded accurate response predictions but unreasonably wide confidence intervals. We therefore used probability-integral-transform (PIT) residual bootstrapping to estimate confidence intervals^34^.

We used the mgcv package^35^ in R^36^ to fit GAMs.

#### Relative infectious burden

In addition to the single-agent time series, we modelled temporal changes in relative infectious burden (RIB), a summary measure of agent loads within a given host^37^. For *a* infective agents assayed in *n* individuals, normalised RIB for individual *i* is:

1 $$\mathop \sum \limits_{j = 1}^{a} \frac{{x_{ij} }}{{a \cdot \mathop {max}\limits_{allk} \left( {x_{kj} } \right)}},$$

where *x*_*ij*_ is infection load (copy number) of for agent *j* in individual *i* (*x*_*kj*_ is the same measure for individual *k*). RIB has been used previously to investigate responses to infection in Chinook salmon^37^ and coho salmon^38^, and it has been characterised in juvenile sockeye salmon^39^.

In calculating RIB, we restricted our dataset to agents for which we had screened in both phases of testing and detected at least five times, plus ASCV and CTV-2, for which we had filled in missing results from the first round of testing (see above). This resulted in RIB calculations based on eighteen infective agents (Table S1).

#### Agent correlations

To assess general patterns of coinfection, we calculated pairwise Spearman rank correlations between assayed copy numbers for pairs of infective agents, data permitting, and between assayed copy numbers and the approximate numbers of house-keeping-gene copies per μL. House-keeping-gene copy number is relevant because degraded host tissue should have a lower number of these gene copies. Because qPCR involves a theoretical doubling of target-DNA copies per cycle, two raised to the power of the negative corresponding Ct value is approximately proportional to the number of target-DNA copies in an assay. In this case, we deemed use of ${2}^{\left({-Ct}_{hkg}\right)}$, ignoring the efficiency of the PCR reaction, to be acceptable, given our use of the measure to calculate a rank correlation.

We used a randomisation test to compare the number of correlations with *p* ≤ 0.05 to the numbers we would expect by chance. In each of 1000 iterations, we independently resampled the data for each agent, with replacement, and re-ran our correlation analysis, as above. While we would expect approximately 19 of the 378 (1/20) correlations to have *p* ≤ 0.05 by chance, this ranomisation test offered a crude comparison to the variation we might expect to see.

## Results

### Data series

#### Infectious-agent data

We successfully generated infectious-agent data for a total of 2931 samples: 2504 from Atlantic salmon farms in the marine environment and 427 from freshwater hatcheries. Of the successfully assayed samples, 2407 were live-sampled fish, while 524 were opportunistically sampled dead or dying fish (160 moribund, 364 recently dead “fresh silvers”). Of the successful hatchery samples, 24 were from dead fish and four were from moribund fish. We initially screened each sample for 47 infectious agents. After screening 933 samples, we replaced assays for eleven extremely rare agents (Table 2) with assays for novel viral variants^30,31^; hence we screened for 58 total agents across the set of samples, but no single fish sample was subjected to every assay. In follow-up screening, we also screened 231 mixed-tissue samples (215 of the initial 933 samples and 16 samples that had not had successful assays previously) for ASCV and CTV-2, the two highest prevalence novel viruses. Overall freshwater agent prevalence ranged from 0 to 39% and overall saltwater prevalence ranged from 0 to 91%, with agents prevalent in freshwater often rare in saltwater, and vice versa (Table 3).

### Statistical analyses

#### Single-agent time series

We note, given the potential bias in the sampling during periods of elevated mortality, that sample trends may not be representative of farm cohorts as a whole.

**Figure 2:**
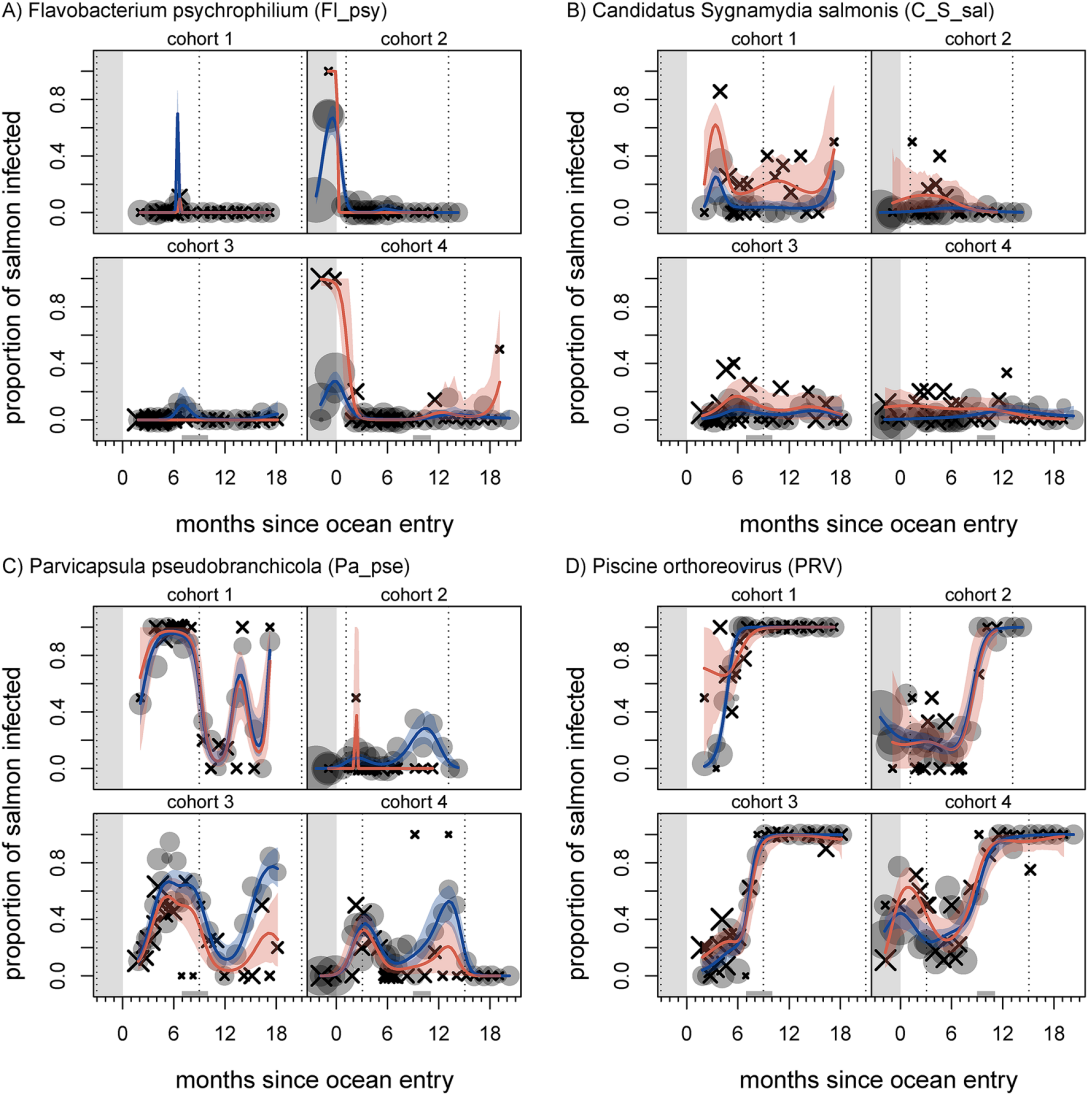
Agent prevalence of *Flavobacterium psychrophilum* (Fl_psy; **A**), salmon gill chlamydia *Candidatus* Syngnamydia salmonis (C_S_sal; **B**), *Parvicapsula pseudobranchicola* (Pa_pse; **C**), and Piscine orthoreovirus (PRV; **D**) in farmed Atlantic salmon throughout four production cycles. Grey circles show prevalence in live fish on each sampling date, and black X’s show prevalence in dead/dying fish (symbol areas proportional to sample sizes). Curves indicate mean predictions from a generalised additive model; blue and red correspond to live and dead/dying fish, respectively (shaded areas show 95% confidence regions). Left-hand grey region indicates freshwater hatchery residence, grey regions on x-axis indicate period of transfer to another site, and vertical dotted lines correspond to January 1st.

In the case of agents for which we had sufficient data to fit descriptive models, prevalence time series generally exhibited one of four patterns: 1) an ephemeral spike around the time of ocean entry followed by decline (*Candidatus* Branchiomonas cysticola, *Flavobacterium psychrophilum* (Fig. 2A), *Vibrio anguillarum*, *Vibrio salmonicida*); 2) low, chronic prevalence, often with an ephemeral marine spike (*Facilispora margolisi*, *Ichthyophthirius multifiliis*, Putative Narna-like virus, *Parvicapsula kabatai*, *Candidatus* Syngnamydia salmonis (Fig. 2B), Putative totivirus); 3) repeatedly fluctuating, substantial prevalence in the marine environment (CTV-2, *Parvicapsula pseudobranchicola* (Fig. 2C), *Tenacibaculum maritimum*); or 4) presence at low levels in freshwater, with increasing prevalence after ocean entry, often to 100% [ASCV, *Kudoa thyrsites*, *Paranucleospora theridion*, Piscine orthoreovirus (PRV; Fig. 2D)]. Figure 2 shows exemplar model fits, with all model fits provided in the Supplementary Information.

**Figure 3:**
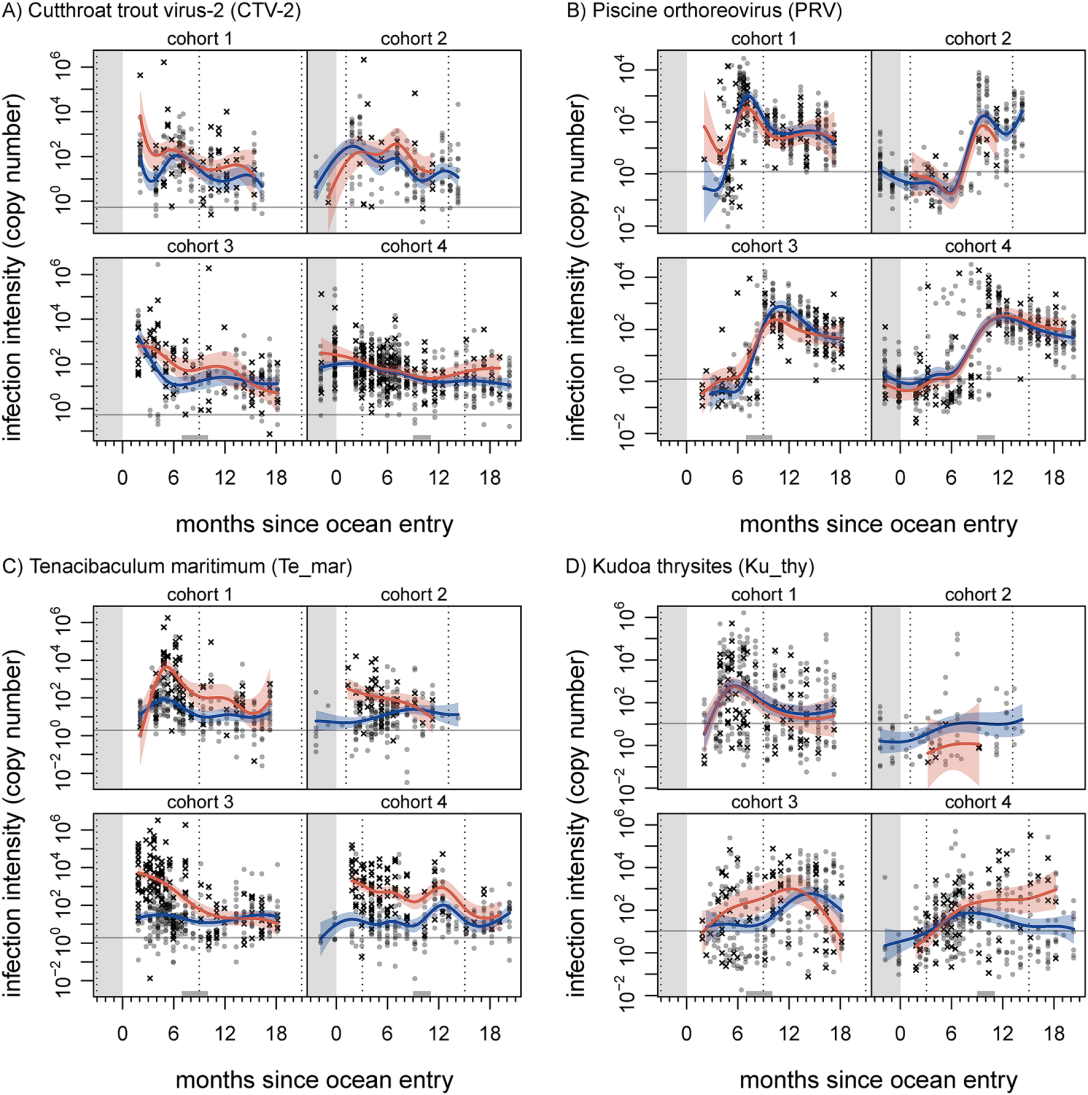
Agent intensity of Cutthroat trout virus (CTV-2; **A**), Piscine orthoreovirus (PRV; **B**), *Tenacibaculum maritimum* (Te_mar; **C**), and *Kudoa thyrsites* (Ku_thy; **D**) in farmed Atlantic salmon throughout four production cycles. Grey circles represent live fish, and black X’s represent dead/dying fish. Curves indicate mean predictions from a generalised additive model; blue and red correspond to live and dead/dying fish, respectively (shaded areas show 95% confidence regions). Left-hand grey region indicates freshwater hatchery residence, grey regions on x-axis indicate period of transfer to another site, and vertical dotted lines correspond to January 1st. Horizontal grey line indicates limit of detection (yielding ≈90% true positive rate) for respective qPCR assay run in duplicate. Note log scale.

Patterns of agent intensity in the available samples were not as well characterised as those for prevalence, in many cases due to sparse data. For many agents, intensity varied by five or more orders of magnitude across individuals and through time. General trends for well-represented agents were: decline in CTV-2 intensity throughout marine residence (Fig. 3A); increase and then decline for PRV (Fig. 3B) and *P. theridion*, both agents that increased in prevalence over time; and substantially elevated but declining intensity for *T. maritimum* in dead and dying fish (Fig. 3C). In many cases, agents for which prevalence plateaued appeared to exhibit an intensity peak at around the time that prevalence approached its maximum (ASCV, *K. thyrsites, P. pseudobranchicola, P. theridion*, PRV; Supplementary Information).

Multiple agents showed differences in prevalence or intensity between live and dead/dying fish. We interpreted models to show statistically significant differences between sample categories in a given time window when the confidence region for one category did not contain the other category’s mean trend. Prevalence of *F. psychrophilum* in dead/dying fish was elevated in-hatchery (Fig. 2A), with intensity also elevated in-hatchery for cohort 4. Intensity of *K. thyrsites* was elevated in dead/dying fish for cohorts 3 and 4 (Fig. 3D). Prevalence of the putative Narna-like virus was elevated in dead/dying fish in cohort 4, although intensities remained around a single gene copy (Fig. S9). In fact, the putative Narna-like virus was particularly prevalent in dead samples (11.9%), compared to dying (1.5%) and live (0.3%) samples. Prevalence of *P. pseudobranchicola* was reduced in dead/dying fish in cohorts 3 and 4 (Fig. 2C). Both prevalence and intensity of PRV were elevated for dead/dying fish shortly after ocean entry in cohort 1 (Figs. 2D, 3B). Prevalence of *T. maritimum* was variously elevated in dead/dying fish, across cohorts, and intensity was also elevated (Figs. 3C, S16), especially in the first year of ocean residence; the latter being perhaps the most striking difference across all agent time series. Prevalence of *C.* Syngnamydia salmonis in dead/dying fish was consistently elevated (Fig. 2B), but did not meet our criterion for significance. Both ASCV and CTV-2 displayed elevated prevalence and intensity in dead/dying fish in cohort 3, just after ocean entry (CTV-2 intensity was also elevated for cohort 1 just after ocean entry; Figs. 3A, S2, S4).

We present comprehensive results for all infectious agents in the supplementary information (Figures S1 through S20).

#### Relative infectious burden

**Figure 4:**
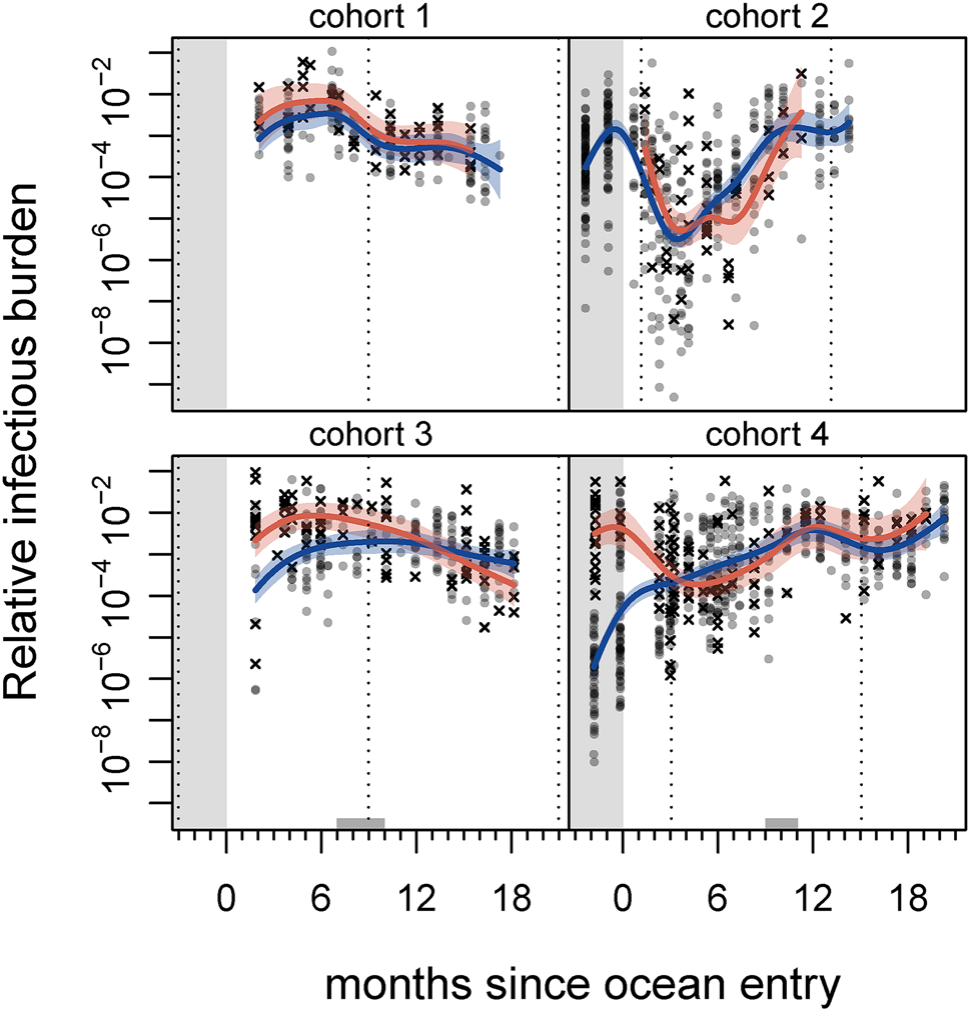
Relative infectious burden (RIB; see main text) multi-agent infection metric in farmed Atlantic salmon throughout four production cycles. Grey circles represent live fish, and black X’s represent dead/dying fish. Curves indicate mean predictions from a generalised additive model; blue and red correspond to live and dead/dying fish, respectively (shaded areas show 95% confidence regions). Grey region indicates freshwater hatchery residence, grey regions on x-axis indicate period of transfer to another site, and vertical dotted lines correspond to January 1st. Note log scale.

Relative infectious burden (RIB) did not show consistent temporal trends across all four sets of samples (Fig. 4). RIB in fish sampled from spring-entry cohorts declined after their first autumn at sea, while RIB in fish sampled from autumn-entry cohorts generally increased after first winter at sea. Cohorts 3 and 4 both showed higher levels of RIB in dead and dying fish prior to their first winter at sea.

#### Agent correlations

**Figure 5:**
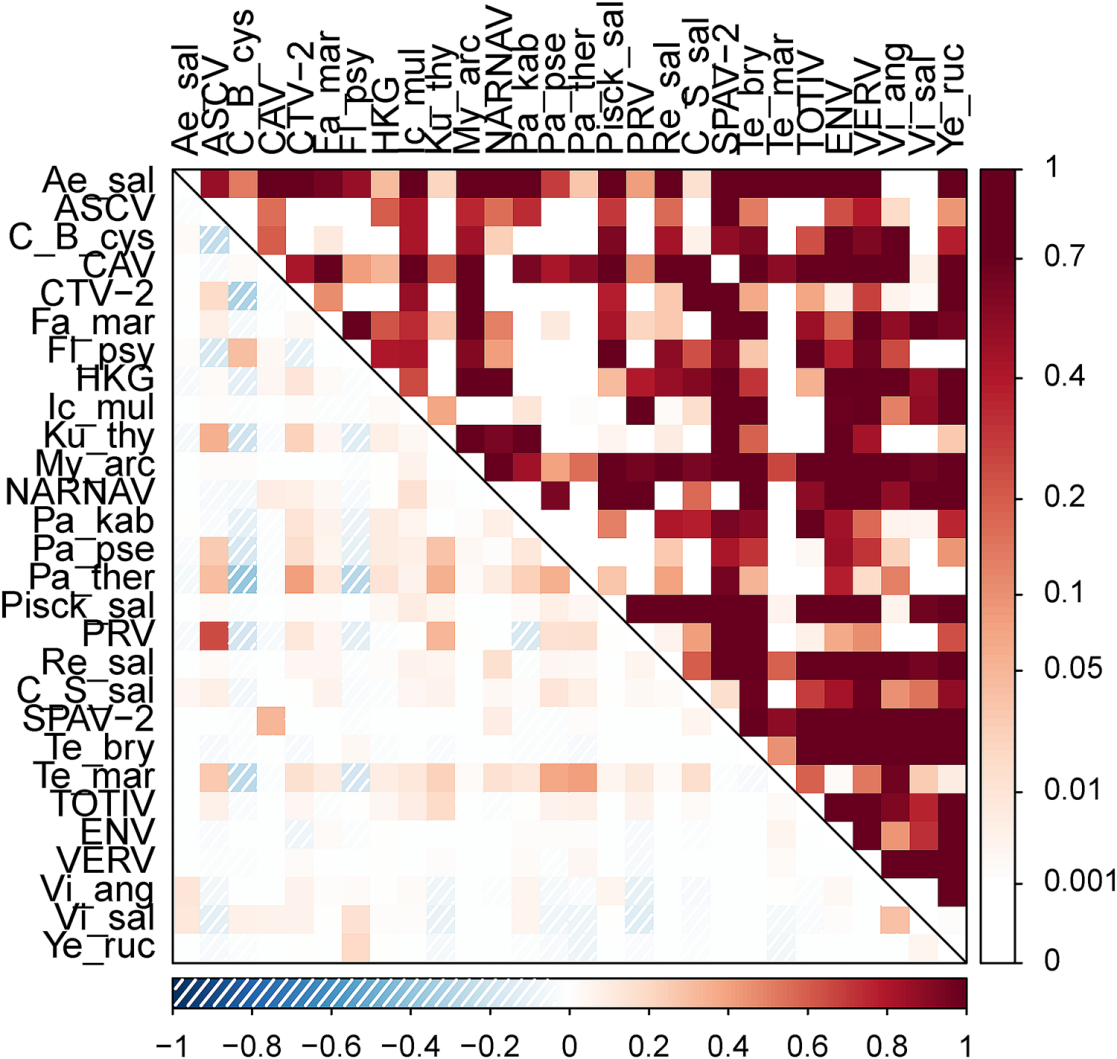
Spearman rank correlations between infectious-agent intensities in farmed Atlantic salmon in BC, Canada throughout four production cycles. See supplementary information for agent abbreviations. Lower left of plot and lower legend indicate correlation values. Upper right of plot and right legend indicate statistical significance of the correlations.

Correlations between infective-agent copy number estimates across all samples ranged from -0.39 (between *P. theridion* & *C.* Branchiomonas cysticola) to 0.65 (between PRV & ASCV). Most correlations were low, and 95% of correlations had an absolute value less than 0.25 (Fig. 5).

Our observed number of correlations with *p* ≤ 0.05 (134) was nearly four times the maximum number of p-values less than 0.05 observed in 1000 randomisation iterations (34; supplementary information).

## Discussion

We used high-throughput qPCR to screen for 58 infective agents in four Atlantic salmon farm cohorts from British Columbia throughout their production cycles. We measured presence and copy number for target genetic sequences, characteristic of specific viral, bacterial, and eukaryotic agents, including several recently discovered viruses^30,31^, known or suspected to cause disease in salmon. These agents displayed various temporal patterns of prevalence and intensity, with several displaying elevated levels in dead and dying fish.

The data and analyses we have presented provide a unique look into the epidemiology of farmed salmon populations, and wildlife/livestock diseases generally. No past studies have had access to multiple farmed-salmon cohorts, throughout their production cycles, with the capacity to molecularly screen for a large suite of infectious agents. Other work has reported agent data for dead-sampled fish collected in BC as part of Fisheries and Oceans Canada’s farm audit program^40^, but such analyses lack the time-series nature of the results we have presented. To our knowledge, no other studies have provided such detailed, comprehensive information for infective agents in domestic or wild populations over time. This study, therefore, presents a substantial step toward effectively monitoring shared wildlife/livestock diseases, made possible by cutting-edge technology, as predicted previously^22^.

While our findings offer specific insight to salmon farmers, aquaculture managers, and those concerned with the disease ecology of sympatric wild salmon, we caution that results remain correlative, and relevant patterns (e.g. apparent mortality signatures) require further investigation. Unfortunately, a lack of regular testing of dead and dying fish (collection was opportunistic, at farms’ discretion) resulted in potential for associated patterns to be obscured. Due to this potential bias in the sampling design, we are unable to draw conclusions related to farm-level mortality rates, but several agents showed patterns of note, including elevated levels in dead and dying fish.

### Agent patterns

Perhaps the clearest single-agent pattern—the elevated load of *T. maritimum* in dead and dying fish (Fig. 3B)—matches generally accepted patterns in aquaculture. Induced tenacibaculosis can be responsible for substantial on-farm mortality worldwide^41^, and mouthrot resulting from *T. maritimum* in the east Pacific causes substantial losses^42^. In our study, mouthrot was noted during veterinarians’ sample processing for cohorts one, three, and four in the months after ocean entry. We note that elevated levels in dead and dying fish could represent the bacterium’s acknowledged role as an opportunistic pathogen^41^, rather than a direct cause of mortality. We also note the positive correlations between *T. maritimum* load and that of a number of different agents (Fig. 5), consistent with the view that *T. maritimum* may facilitate co-infections^43^. Given its high overall prevalence in fish (Table 3), secondary factors—such as co-infections—might exacerbate infection with *T. maritimum*, playing a role in mortality.

*K. thyrsites intensity* was elevated in dead and dying fish for cohorts three and four, around the time they were transferred to their final marine locations (Fig. S8). In both cases, the cohorts finished their production cycles in farms on the central east coast of Vancouver Island (Fig. 1), a region in which the risk of *K. thyrsites* infection is acknowledged to be high^44^. This myxozoan parasite is economically important due to post-mortem myoliquefaction seen in infected fish, but it is not considered a pathogen^45^, and it is unclear why higher gene-copy levels would be observed in dead/dying fish. *K. thyrsites* may merely replicate faster in stressed fish (in this case due to transport). Our surveillance of pathogens did not include skeletal muscle tissue, where *K. thyrsites* spores develop, but it did include heart, which can be infected by the parasite^46^. We note that *K. thyrsites* was correlated with PRV, with both agents known to infect muscle tissue (although red blood cells are the primary infective tissue for PRV). Follow-up histopathological investigation may provide some insight into *K. thyrsites* distribution and any associations with pathology or patterns of co-infection.

PRV, which is the causative agent of Heart and Skeletal Muscle Inflammation (HSMI)^47^ and has recently generated controversy in BC^28,29,48^, shows several patterns of note. PRV prevalence increased to near ubiquity over time (Fig. 2D), concurrent with an increase, peak, slight decline, and then stabilisation of intensity (Fig. 3B). Although our perspective is limited to sampled fish, with a noted potential for bias, the observed PRV trends were consistent across all four cohorts, and the intensity patterns are consistent with previously reported dissemination, peak replication, and long-term persistence phases of the virus within hosts^29,48^. Past findings suggest that PRV may induce an antiviral response in hosts that can protect them against certain co-infections^49,50^. Perhaps counter to the generality of this claim, PRV and ASCV exhibited the strongest load correlation out of any we observed across our data set (Fig. 4). ASCV was originally isolated from salmon with HSMI, and was initially thought to play a role in the disease^51^. Other work has found no relationship between ASCV and PRV infections or HSMI^52^. In the case of a related baitfish calicivirus, however, there is evidence that viral co-infection is linked to disease manifestation^53^, so further work is needed to tease these relationships apart. In general, dead and dying Atlantic salmon in our study did not show elevated prevalence or intensity of PRV, except shortly after ocean entry in cohort one (Figs. 2D, 3B). This mortality signature corresponds to the onset of lesions diagnostic of HSMI in cohort one, which subsequently spread to affect the majority of that farm population for most of a year^29^.

The gill chlamydia bacterium, *C.* Syngnamydia salmonis, showed a consistent trend towards elevated prevalence in dead and dying salmon (Fig. 2B). Observed intensity was low, however, often averaging approximately a single copy (Fig. S15). Sequencing has validated past detections of this agent on the Fluidigm BioMark™, and has also revealed SNP diversity within the primer-binding region, resulting in potential underdetection (Miller et al. unpublished). Given a putative mortality signature and the lack of prior epidemiological study of this recently discovered agent^54,55^, we would suggest further work on *C.* Syngnamydia salmonis.

Ephemeral mortality signatures appeared for several agents. *F. psychrophilum* was clearly elevated in dead and dying fish in-hatchery, although we only had access to two hatchery cohorts and cannot draw general conclusions. Intensity of both the ASCV and CTV-2 were elevated in sampled dead and dying fish from at least one cohort shortly after ocean entry (Figs. 3A, S2). Both viruses were also present in-hatchery. The previously reported Cutthroat trout virus appears to be apathogenic^56^ in trout, and has been detected in Atlantic salmon^57^. Little is known about the novel variant for which we screened, although in situ hybridisation has revealed that infection can be systemic and extensive in the brain (Mordecai et al. 2020). As for the ASCV, associated pathology was found to be likely due to PRV contamination^51^. Extremely limited information about these two viruses, paired with our findings, warrants further investigation (e.g. with histopathology and in situ hybridisation) to determine if either virus is linked with pathology. As both these viruses were detected in Chinook salmon (Mordecai et al. 2020), and considering their high prevalence in Atlantic salmon farms, the potential risk they pose to wild Pacific salmon populations should be a priority for future research.

Infectious agent levels overall, as measured by relative infectious burden, showed a clear trend in smolts coming out of freshwater hatcheries for cohorts three and four. There, infectious burden was much higher (in one case 10 000 times higher) in dead and dying fish than in live-sampled fish. While the effect dissipated once fish entered the marine environment, it is clear that hatchery fish are dying with—or of—elevated levels of infection. The patterns we observed likely reflect the transition from freshwater to saltwater, with a coincident shift in infective-agent communities^58^. Smoltification has also been associated with immune depression^59^, and elevated infectious burden around the time of ocean entry may reflect this. Where we had dead/dying hatchery samples, however, infectious burden was elevated weeks before ocean entry, hinting at the potential for problems in-hatchery.

Across all agents, we observed apparent coinfection signals that clearly differed from random chance (Figs. 5, S21). We point out, however, that due to the longitudinal nature of our study and cursory investigation of agent correlations, the correlation results almost certainly indicate shared temporal trends that may or may not indicate underlying interactions. For example, *Candidatus* Branchiomonas cysticola and *Flavobacterium psychrophilum* were positively correlated with each other, but negatively correlated with a number of other agents. This could be due to both agents being common in-hatchery but not in the marine environment (Figs. S3, S6), counter to many other agents’ patterns. Alternatively, the correlation could be due to a biological relationship, perhaps in relation to gill health. We intend to follow up on a number of correlational patterns.

### Agent idiosyncrasies

Several agents showed unexpected patterns, or patterns that may be connected to their particular biology.

The putative Narna-like virus, a recently discovered agent^31^, showed elevated prevalence in dead and dying fish (Fig. S9). This pattern was mainly due to over-representation in dead-sampled fish, as we detected the agent in 13.2% of dead fish, 1.6% of moribund fish, and 0.4% of live-sampled fish in saltwater. Given that *Narnaviridae*, of which the putative Narna-like virus is a member, is thought mainly to infect fungi^60^, this virus may be associated with a fungal decomposer. This is speculative, however, and recent genomic evidence from across taxa suggests that the *Narnaviridae* may be much more widespread than previously thought^61^.

Counter to the common trend, *P. pseudobranchicola* tended to be less common in dead and dying fish than in live-sampled fish (Fig. 2C), with dead fish, in particular, tending to exhibit the lowest levels (results not shown). *P. pseudobranchicola* primarily infects the pseudobranch^62^, a structure near the gills involved in oxygenating blood in the eye. Infection also occurs in tissue collected for this study, especially gill^63^, and we speculate that loads in dead fish could be reduced due to myxospore release or degradation of delicate gill tissue after host death. Given that we did not sample the pseudobranch, it is likely that our data underestimates the load of this organism.

The sampling environments (freshwater or marine) of several detections were unexpected. In particular, we detected *K. thyrsites* and *T. maritimum* (Fig. 3C) in freshwater hatcheries, although these agents are considered marine species^64,65^. It is possible that these hatcheries introduced saltwater in the weeks before ocean transfer, to prepare smolts for release. We also detected *F. psychrophilum,* considered a freshwater bacterium^66^, in marine net pens (Fig. 2A). The bacterium is known to survive in brackish water^67^, however, and this is not the first time it has been detected in a marine setting^40,68^.

### Broader connections

Not all infective agents cause disease, and even agents that do can be present long before—or long after—clinical symptoms. Our work presents only a piece of the puzzle in what is a multifaceted, complex scenario of shared wildlife/livestock disease in salmon aquaculture. The controversy surrounding PRV in BC, as an example, illustrates this complexity. While conventional lab challenges using PRV from BC sources have failed to reproduce in BC fish the extent of HSMI lesions observed on Norwegian farms^48,69^, work related to our study has been able to identify and shed light on HSMI, and related jaundice/anemia in Chinook salmon, in BC salmon farms^28,29^. While we saw a putative mortality signature in one cohort during this study, the normal course of PRV infection was not always associated with mortality (e.g. Figs. 2D, 3B). More work will be required to elucidate the nuances of PRV infection, factors that induce associated disease, and possible resultant mortality. A fruitful place to start would be to carry out sampling and diagnostics of dead and dying fish in farms and pens experiencing elevated mortality.

Although we have shown putative mortality signatures for several infective agents in farmed Atlantic salmon, these are not necessarily the agents that pose the greatest risk to wild salmon. For one thing, a given agent need not produce the same effects in different species^28,70^. For another, contact between populations may not coincide with infection maxima. Depending on when farm smolts enter the marine environment, for example, PRV could be at low prevalence in the spring, when a number of wild Pacific salmon species migrate as juveniles^15^. Other times of year would be more relevant for interactions with other wild species, and there is much scope for transfer between farmed and wild environments. In addressing shared wildlife/livestock disease, we need to consider both wildlife and livestock as populations that serve as potential reservoirs of disease agents, and are susceptible to outbreaks^71^. In this context, surveillance and monitoring are essential facets of disease management^23^, providing raw material to develop understanding of disease and build effective management strategies. Parallel work is monitoring wild populations for the same agents we have investigated here, with the prospect of cross-referencing patterns and impacts^72–74^.

The ubiquity of infectious agents on the farms leads naturally to discussion of potential control strategies, which present a variety of challenges in aquaculture. Vaccination has proven successful at times, but the salmon aquaculture industry has a somewhat chequered history with uptake, since vaccination can affect host growth, and thus the bottom line^13^. In addition, vaccines have only been developed for a handful of agents. Reducing translocations can be an effective control strategy on land^20,78^, but transmissive properties of the marine environment and highly mobile marine carrier hosts pose challenges to isolating host populations geographically (Krkosek 2015). Our findings provide circumstantial evidence that some agents (e.g. *K. thyrsites*) respond to translocations. The fact that two of our four focal cohorts moved substantial distances throughout their respective marine production may be cause for concern, considering the infective-agent populations we have shown those cohorts to have harboured. In general, aquaculture-associated disease and related management decisions have a history of generating political controversy^75^. Infective-agent monitoring and analyses are critical for designing, implementing, and evaluating effective disease-control measures, and for bridging divides in debate surrounding aquaculture.

With respect to the aquaculture industry, the tools we employed in this study may prove useful for disease management and fish health. We have shown that for many agents, patterns of infection in dead and dying fish mirror those in live fish. By integrating high-throughput infectious-agent screening with existing monitoring of dead fish, farm vets and managers could access a wealth of otherwise unavailable or costly information. Combining such results with strategic sacrificial sampling of live fish during mortality peaks could allow additional insight into which agents may be driving mortality. Protecting the ‘herd’ (and its wild neighbours) may justify such mortal sampling. Furthermore, in other ongoing work, we have seen that much infective-agent information is accessible via nonlethal gill biopsy, which also enables high-throughput screening for gene expression patterns associated with various patterns of stress^76^ and disease^77^. Used appropriately, such a combination of tools could be very powerful.

Disease monitoring is never complete, and detection always lags behind pathogen spread^78^, but new technologies—such as those we employed here – can facilitate efficient, lower-cost surveillance and monitoring. Surveillance for existing pathogens and identification of previously unknown pathogens is part of the integrative approach required to understand and control existing and emerging infectious diseases^22^. Here, we have further demonstrated the utility of high-throughput, modern genetic techniques for monitoring known infective agents and for generating information about previously under-studied agents^26,29–31,37,38^. Further work will target the risk of transfer between wild and farmed hosts and prioritize threats to salmon, farmed and wild.

## Supplementary Information

Supplementary Information.

## Data Availability

Data are available on the Dryad data repository at 10.5061/dryad.x95x69pjz.
