# Author Correction: Descriptive multi-agent epidemiology via molecular screening on Atlantic salmon farms in the northeast Pacific Ocean

**DOI:** 10.1038/s41598-021-01421-0

**Published:** 2021-11-01

**Authors:** Andrew W. Bateman, Angela D. Schulze, Karia H. Kaukinen, Amy Tabata, Gideon Mordecai, Kelsey Flynn, Arthur Bass, Emiliano Di Cicco, Kristina M. Miller

**Affiliations:** 1 Pacific Salmon Foundation, Vancouver, Canada; 2 Ecology and Evolutionary Biology, University of Toronto, Toronto, Canada; 3 Molecular Genetics, Fisheries and Oceans Canada, Nanaimo, Canada; 4 Department of Medicine, University of British Columbia, Vancouver, Canada; 5 Forest and Conservation Sciences, University of British Columbia, Vancouver, Canada

Correction to: *Scientific Reports* https://doi.org/10.1038/s41598-020-78978-9, published online 10 February 2021

The original version of this Article contained errors.

In Table 1, the cohort 2 “Hatchery” Exit and “Farm” Entry months were inadvertently switched with the “Farm” Exit month. The correct and incorrect values appear below.

Incorrect:

**Table:** 

cohort	Facility type	Entry (initial)	Exit (final)
2	Hatchery	-	Sep 2013
	Farm	Sep 2013	Nov 2015

Correct:

**Table:** 

cohort	Facility type	Entry (initial)	Exit (final)
2	Hatchery	-	Nov 2013
	Farm	Nov 2013	Sep 2015

Additionally, the Data Availability section was incorrect.

“Data will be made available in UBC’s Strait of Georgia Data Centre (sogdatacentre.ca) prior to publication.”

now reads:

“Data are available on the Dryad data repository at https://doi.org/10.5061/dryad.x95x69pjz.”

The original Article has been corrected.

