# Supplementary material for: Descriptive multi-agent epidemiology via molecular screening on Atlantic salmon farms in the northeast Pacific Ocean
Source: Sci Rep. 2021 Feb 10;11:3466. doi: 10.1038/s41598-020-78978-9 (PMC7876018; doi:10.1038/s41598-020-78978-9)
Supplement: Supplementary file 1 — Supplementary Information. [file 41598_2020_78978_MOESM1_ESM.pdf]

Supplemental information for:
Descriptive multi-agent epidemiology via molecular screening on four Atlantic salmon farms in the northeast Pacific Ocean

Andrew W. Bateman, Angela D. Schulze, Karia H. Kaukinen, Amy Tabata, Gideon Mordecai, Kelsey Flynn, Arthur Bass, Emiliano Di Cicco, Krisitina M. Miller

Supplemental methods: laboratory details

To extract nucleic acid for testing, samples were homogenized in a Mixer Mill (Qiagen, Maryland, USA) using Tri-reagent™ (Ambion Inc., Austin, TX, USA). After adding 1-bromo-3-chloropropane to the homogenate and centrifugation, aliquots of the aqueous phase (for RNA) and organic/interphase (for DNA) were pipetted separately into 96-well plates. Total RNA was extracted using the Magmax™-96 for Microarrays RNA kit (Ambion Inc, Austin, TX, USA) with a Biomek NXP™ (Beckman-Coulter, Mississauga, ON, Canada) automated liquid handling instrument, using the "spin method" protocol. RNA was reverse transcribed into cDNA using the superscript VILO master mix kit (Invitrogen, Carlsbad, CA). DNA was extracted from the organic/interphase layer of the Tri-reagent™ homogenate using the TNES-6U method, following the Qiagen BioSprint protocol. Nucleic acid quantities were determined using spectrophotometry and then normalised to 62.5 ng/µL using a Biomek NXP™. We performed all procedures with commercial kits and hardware based on manufacturers' instructions.

After extraction, we subjected DNA and cDNA to a Specific Target Amplification (STA) step, amplifying target DNA sequences by a known factor, to aid target quantification during nanolitre-volume BioMark™ reactions. While we did not multiplex assays on the BioMark™, the STA (run on a conventional PCR machine) did involve multiplexing low concentrations (1/20th of normal concentrations) of all primers to be run on a single dynamic array. The 5 µL STA reaction contained 1.3 µL of cDNA/DNA, 1X TaqMan PreAmp master mix (Applied Biosystems, Foster City, CA, USA), and 0.2 µM of each primer. The 14-cycle STA program was run according to manufacturer's instructions for TaqMan gene expression assays (Fluidigm Corporation, South San Francisco, CA, USA). Upon completion of the STA, excess primers were removed by treating with Exo-SAP-IT™ (no interference in downstream application, 100% recovery of PCR products; Affymetrix, Santa Clara, CA, USA) according to manufacturer's instructions and then diluted 1/5 in DNA re-suspension buffer (Teknova, Hollister, CA, USA).

The BioMark™ 96-by-96 gene-expression dynamic arrays (Fluidigm Corporation, South San Francisco, CA, USA) were run according to the procedure described elsewhere (Miller et al. 2016). For each sample, we prepared 5 µL of template mixture containing 1 × TaqMan Universal Master Mix (No UNG), 1 × GE Sample Loading Reagent (Fluidigm PN 85000746), and the corresponding normalised and pre-amplified sample mixture. We prepared 5 µL of each assay mixture with 1 × the appropriate TaqMan qPCR assay (including 9 µM of primers and 2 µM of agent probe in FAM-MGB and 1.2 µM of artificial probe construct [APC] in NED-MGB) and 1 × Assay Loading Reagent (Fluidigm PN 85000736). We added controls prior to running the dynamic array. APC clone targets for all assays were contained in a single serially diluted set of pools, loaded last, minimizing the likelihood of contamination of any single APC clone. Once loading and mixing of the dynamic array were completed within the IFC HX controller, the array was transferred to the BioMark™ HD instrument and processed using the GE 96x96

Standard TaqMan program for qPCR, which includes a hot start followed by 40 cycles at 95°C for 15 s and 60°C for 60 s (Fluidigm Corporation, South San Francisco, CA, USA).

We used pooled-tissue cDNA as the template for Atlantic Salmon Calicivirus and Cutthroat Trout Virus quantification using an ABI 7900HT (Applied Biosystems) in 384-well optical plates. The qPCR reaction volume was 12 mL, which comprised 6 mL of 2XTaqMan Gene Expression Master Mix (Applied Biosystems PN 4369016), 4.3 mL of water, 0.22 mL of mixed forward and reverse primers (900 nM final concentration of each), 0.24 mL of each probe (200 nM final concentration; assay specific probe and APC control probe), and 1 mL of cDNA template. Temperature cycles included one 2 min hold (50°C), a 10 min denaturation (95°C), and 40 cycles of denaturation (95°C for 15 s), annealing and extension (60°C for 60 s). Amplification conditions on the ABI 7900 were not optimised for this platform, but rather closely reflected those used on the BioMark platform. Samples run on the ABI did not undergo STA enrichment. Standard curves were constructed using single assay APC clone standards spiked in with CHSE DNA as on the BioMark. Serial dilutions clone standards, unknown samples, and positive and negative controls were all run in duplicate..

We processed the BioMark™ results with Real-Time PCR Analysis Software (Fluidigm Corporation, CA, USA). Initially, this software fits curves relating the cycle at which each standard dilution crosses a given threshold (its Ct value) to its known DNA-target concentration. Finally, these curves and the sample Ct values yielded the approximate number of copies of each nucleic-acid assay target per µL of normalised nucleic-acid solution, prior to STA-amplified. Similarly, we used ABI software to calculate copy numbers based on standard curves.

Supplemental results

Table S1. Infective agents used in calculation of relative infectious burden (restricted to agents screened in both rounds with at least five detections, as well as ascv and ctv-2, for which fill-in screening was performed).

test	organism
ASCV	Atlantic Salmon Calicivirus
CTV-2	Cutthroat Trout Virus
PRV	Piscine orthoreovirus
ENV	Erythrocytic necrosis virus
Ae_sal	*Aeromonas salmonicida*
C_B_cys	*Candidatus Branchiomonas cysticola*
Fl_psy	*Flavobacterium psychrophilum*
C_S_sal	*Candidatus* Syngnamydia salmonis

Te_mar	*Tenacibaculum maritimum*
Vi_ang	*Vibrio anguillarum*
Vi_sal	*Vibrio salmonicida*
Ye_ruc	*Yersinia ruckeri*
Fa_mar	*Facilispora margolisi*
Pa_ther	*Paranucleospora theridion*
ku_thy	*Kudoa thyrsites*
Pa_kab	*Parvicapsula kabatai*
Pa_pse	*Parvicapsula pseudobranchicola*
Ic_mul	*Ichthyophthirius multifiliis*

Note: in the figures below, “limit of detection” (LoD) refers to the number of target sequence copies per µL that, during validation studies (Miller et al. 2016 and equivalent calculations for more recently developed assays), yielded a 95% true-positive detection rate. The use of paired assays in this study, where both assays must agree, means that ≈90% (0.95*0.95) of assays would yield positive results at the LoD concentration. Concentrations of target nucleic acid below the LoD concentration will less often result in detections.

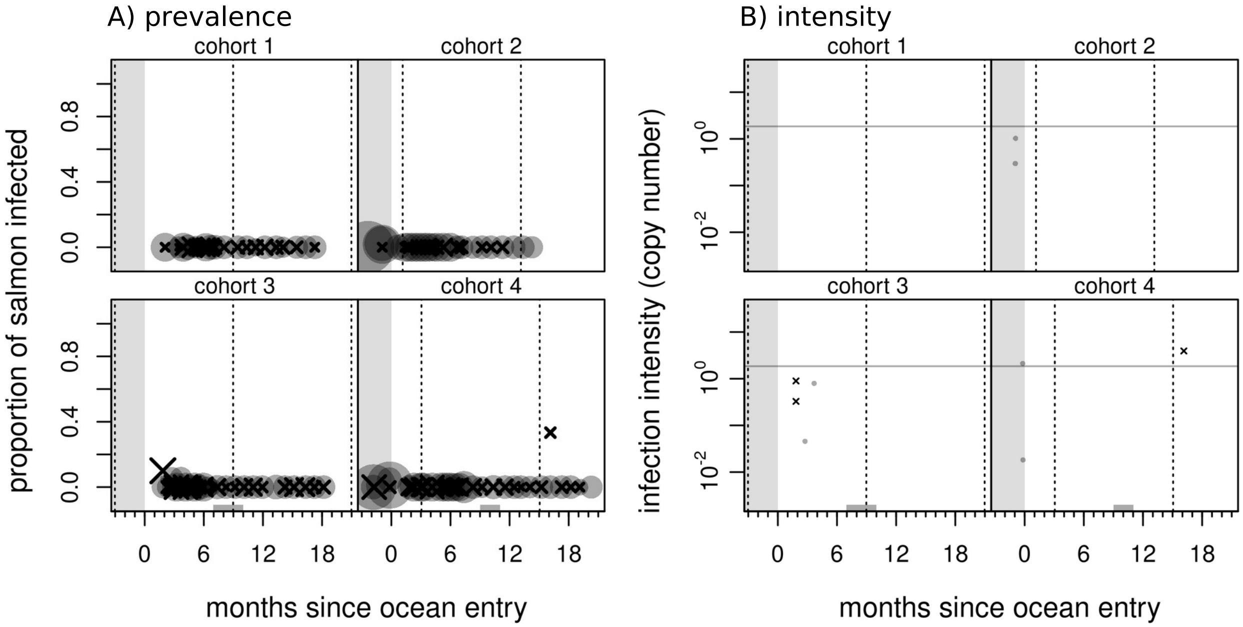

Figure S1. *Aeromonas salmonicida* (Ae_sal) prevalence (A) and intensity (B) in farmed Atlantic salmon throughout four production cycles. Grey circles and black X’s represent live and dead/dying fish, respectively (symbol area proportional to sample size for daily prevalences). Left-hand grey region shows hatchery residence, grey regions on x-axis indicate period of transfer to another site, and vertical dotted lines show January 1st. Horizontal grey line indicates limit of detection. Note different scales.

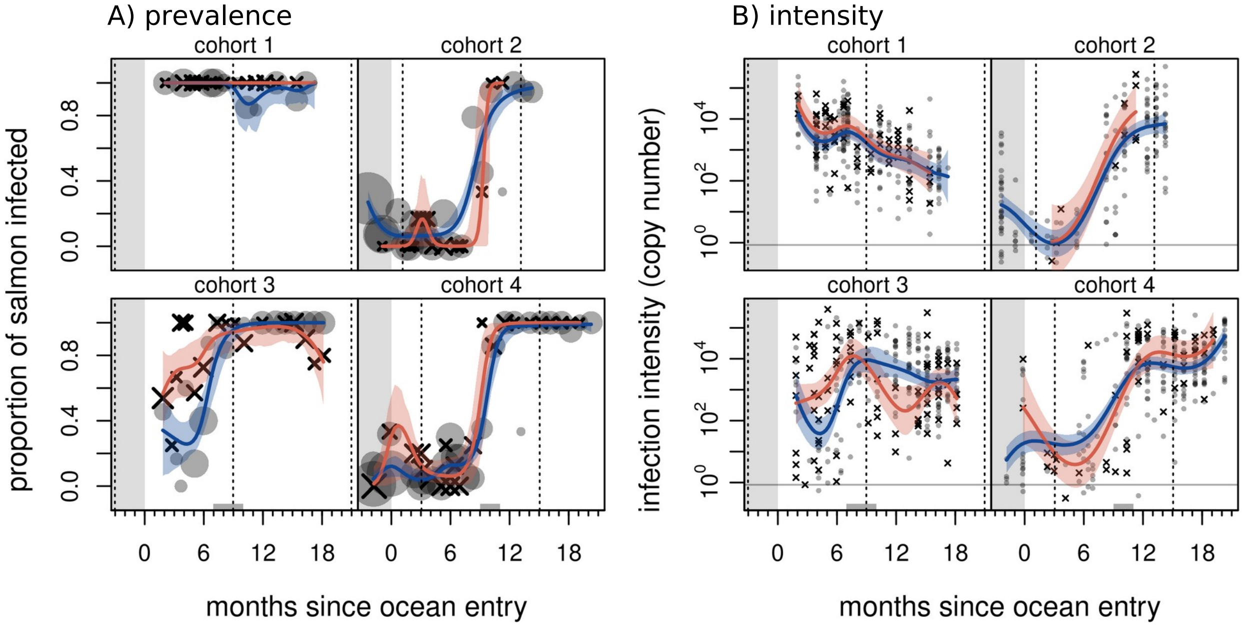

Figure S2. Atlantic Salmon Calicivirus (ASCV) prevalence (A) and intensity (B) in farmed Atlantic salmon throughout four production cycles. Grey circles and black X's represent live and dead/dying fish, respectively (symbol area proportional to sample size for daily prevalences). Mean curves and 95% confidence regions from generalised additive models (blue: live, red: dead/dying). Left-hand grey region shows hatchery residence, grey regions on x-axis indicate period of transfer to another site, and vertical dotted lines show January 1st. Horizontal grey line indicates limit of detection. Note different scales.

A) prevalence B) intensity

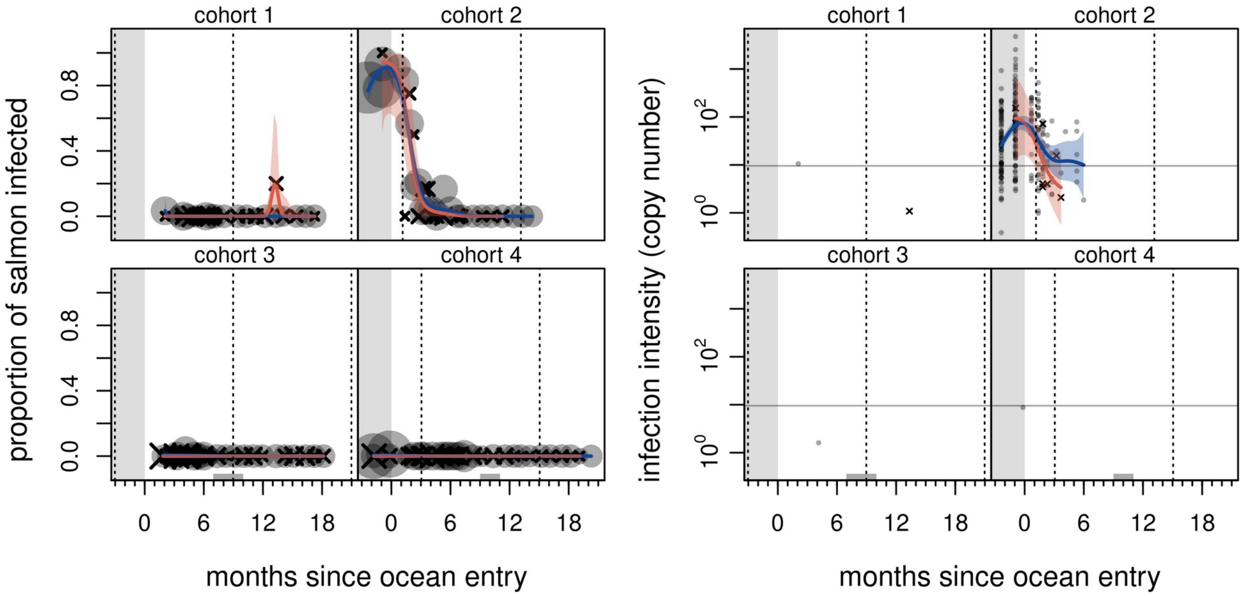

Figure S3. *Candidatus* Branchiomonas cysticola (C_B_cys) prevalence (A) and intensity (B) in farmed Atlantic salmon throughout four production cycles. Grey circles and black X's represent live and dead/dying fish, respectively (symbol area proportional to sample size for daily prevalences). Mean curves and 95% confidence regions from generalised additive models (blue: live, red: dead/dying). Left-hand grey region shows hatchery residence, grey regions on x-axis indicate period of transfer to another site, and vertical dotted lines show January 1st. Horizontal grey line indicates limit of detection. Note different scales.

A) prevalence B) intensity

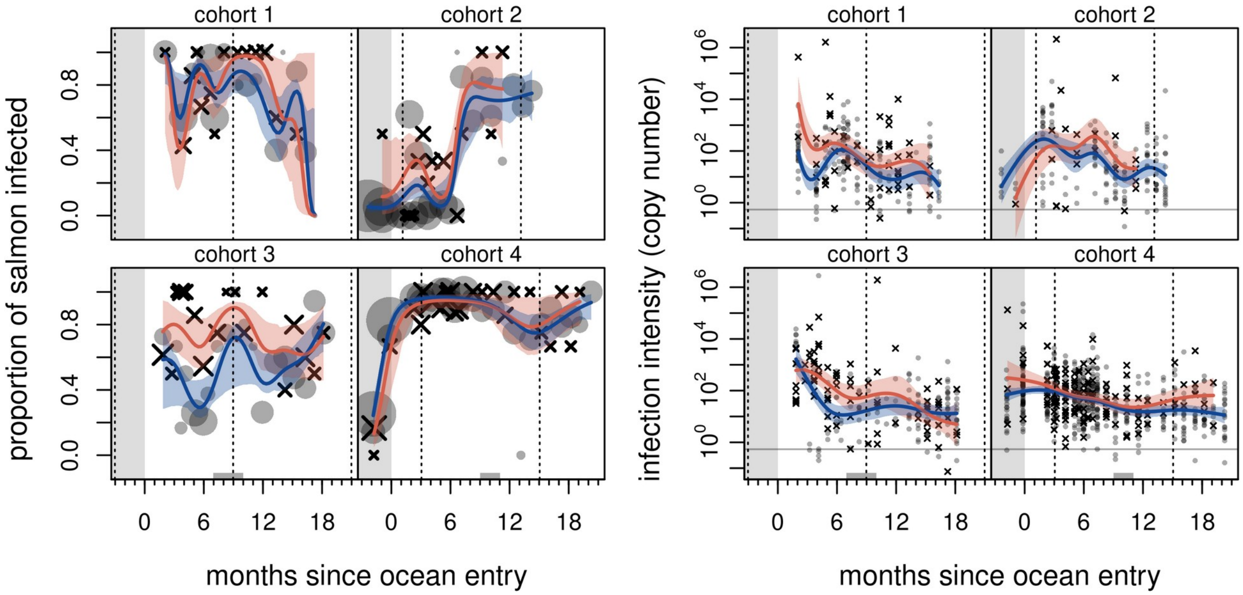

Figure S4. Cutthroat Trout Virus strain 2 (CTV-2) prevalence (A) and intensity (B) in farmed Atlantic salmon throughout four production cycles. Grey circles and black X's represent live and dead/dying fish, respectively (symbol area proportional to sample size for daily prevalences). Mean curves and 95% confidence regions from generalised additive models (blue: live, red: dead/dying). Left-hand grey region shows hatchery residence, grey regions on x-axis indicate period of transfer to another site, and vertical dotted lines show January 1st. Horizontal grey line indicates limit of detection. Note different scales.

A) prevalence B) intensity

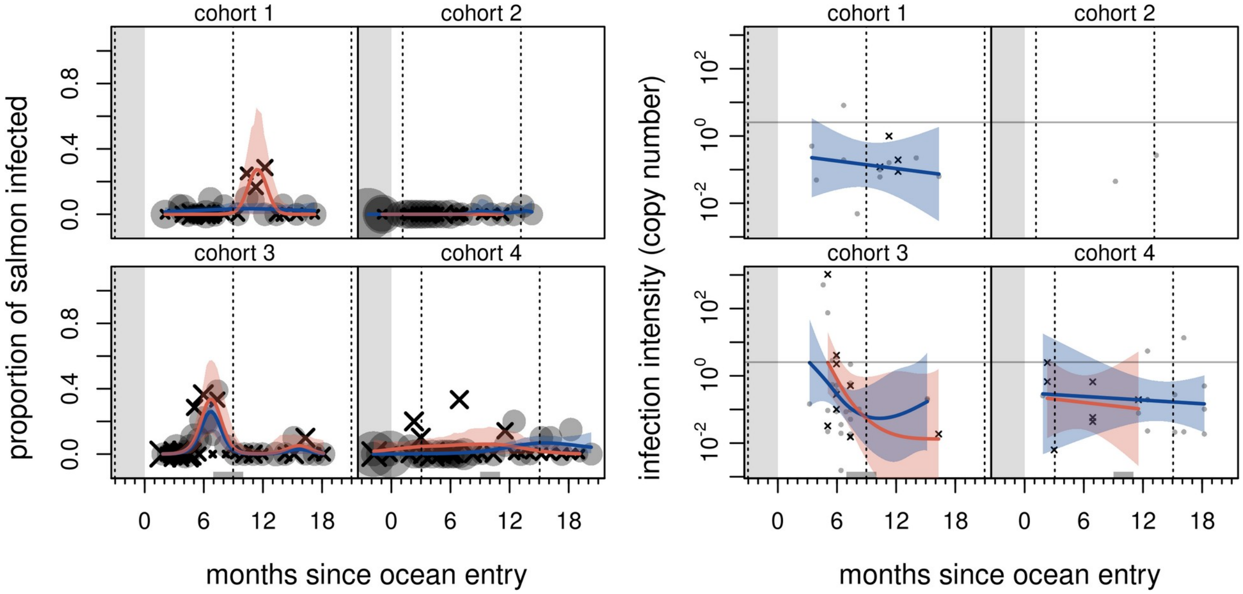

Figure S5. *Facilispora margolisi* (Fa_mar) prevalence (A) and intensity (B) in farmed Atlantic salmon throughout four production cycles. Grey circles and black X's represent live and dead/dying fish, respectively (symbol area proportional to sample size for daily prevalences). Mean curves and 95% confidence regions from generalised additive models (blue: live, red: dead/dying). Left-hand grey region shows hatchery residence, grey regions on x-axis indicate period of transfer to another site, and vertical dotted lines show January 1st. Horizontal grey line indicates limit of detection. Note different scales.

A) prevalence B) intensity

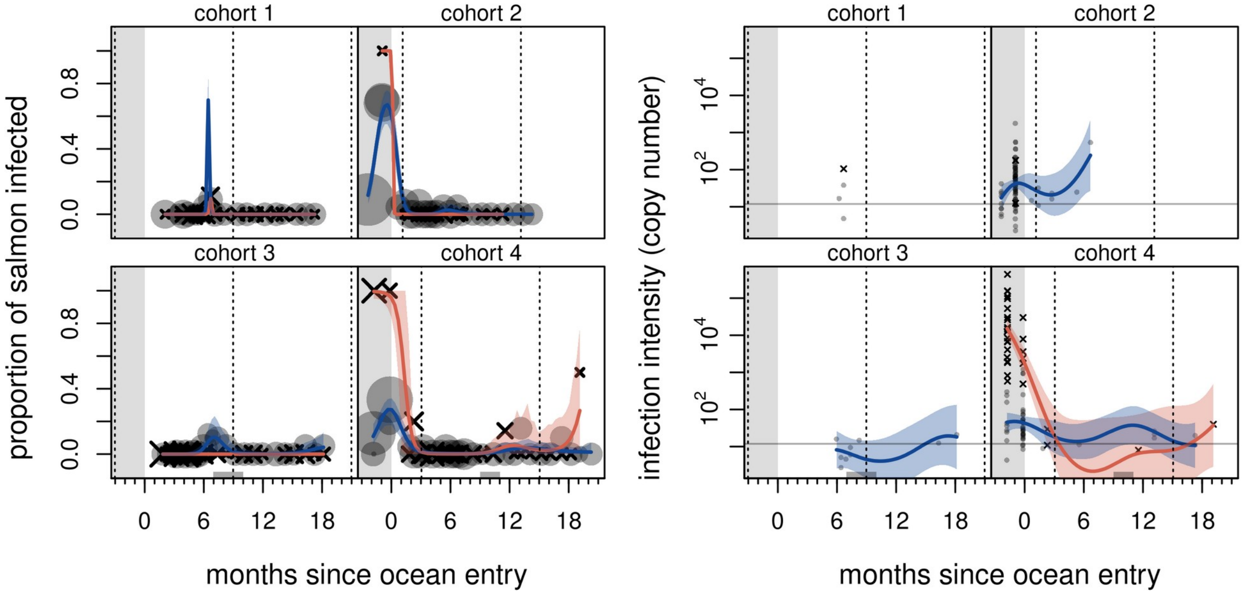

Figure S6. *Flavobacterium psychrophilum* (Fl_psy) prevalence (A) and intensity (B) in farmed Atlantic salmon throughout four production cycles. Grey circles and black X's represent live and dead/dying fish, respectively (symbol area proportional to sample size for daily prevalences). Mean curves and 95% confidence regions from generalised additive models (blue: live, red: dead/dying). Left-hand grey region shows hatchery residence, grey regions on x-axis indicate period of transfer to another site, and vertical dotted lines show January 1st. Horizontal grey line indicates limit of detection. Note different scales.

A) prevalence B) intensity

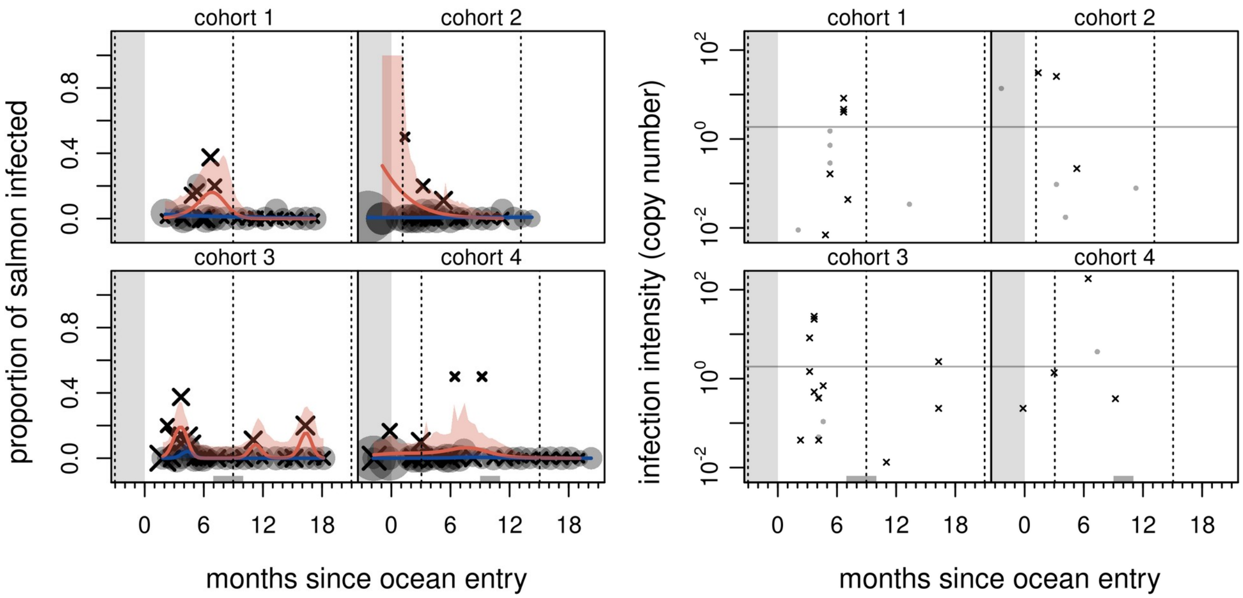

Figure S7. *Ichthyophthirius multifiliis* (Ic_mul) prevalence (A) and intensity (B) in farmed Atlantic salmon throughout four production cycles. Grey circles and black X's represent live and dead/dying fish, respectively (symbol area proportional to sample size for daily prevalences). Mean curves and 95% confidence regions from generalised additive models (blue: live, red: dead/dying). Left-hand grey region shows hatchery residence, grey regions on x-axis indicate period of transfer to another site, and vertical dotted lines show January 1st. Horizontal grey line indicates limit of detection. Note different scales.

A) prevalence B) intensity

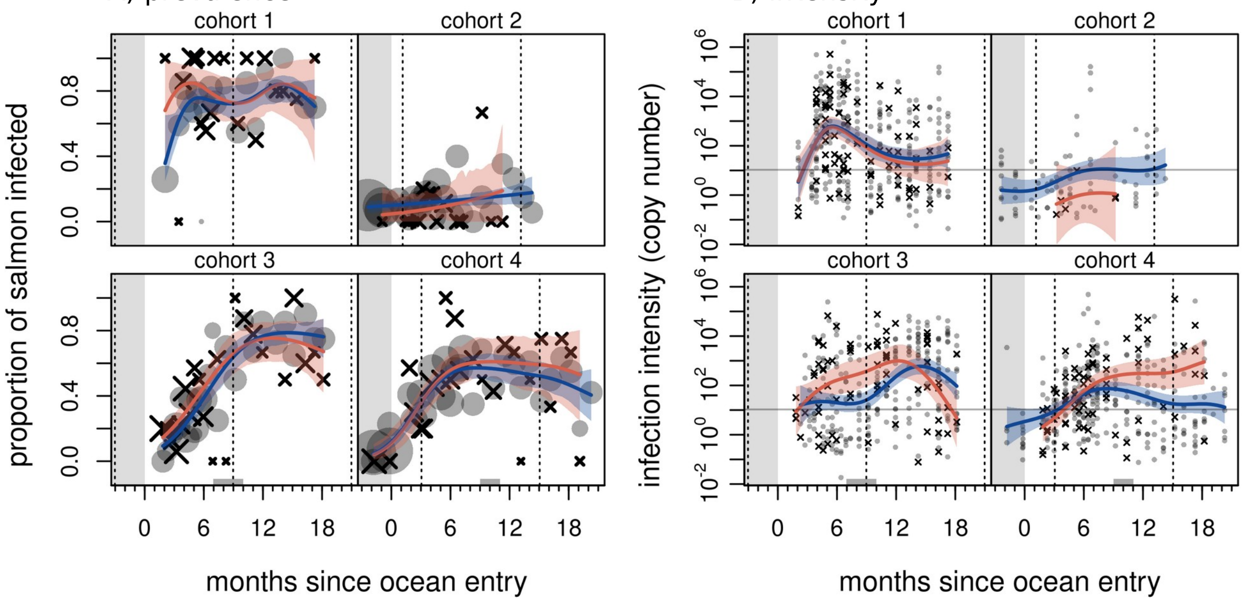

Figure S8. *Kudoa thyrsites* (Ku_thy) prevalence (A) and intensity (B) in farmed Atlantic salmon throughout four production cycles. Grey circles and black X's represent live and dead/dying fish, respectively (symbol area proportional to sample size for daily prevalences). Mean curves and 95% confidence regions from generalised additive models (blue: live, red: dead/dying). Left-hand grey region shows hatchery residence, grey regions on x-axis indicate period of transfer to another site, and vertical dotted lines show January 1st. Horizontal grey line indicates limit of detection. Note different scales.

A) prevalence B) intensity

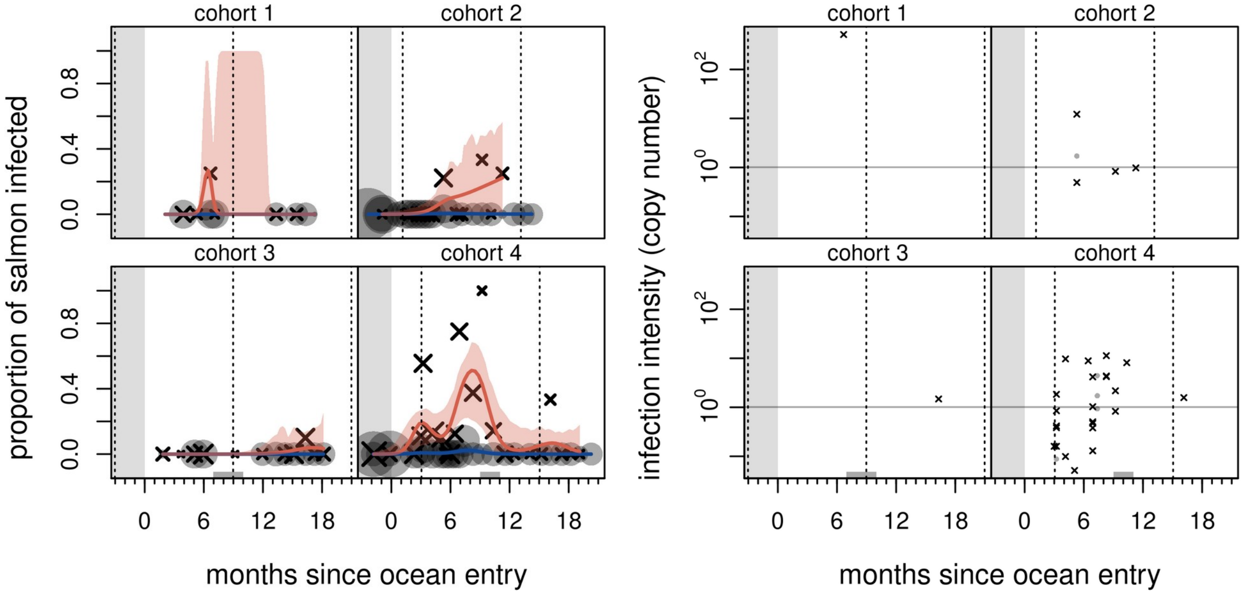

Figure S9. Putative Narna-like virus (NARNAV) prevalence (A) and intensity (B) in farmed Atlantic salmon throughout four production cycles. Grey circles and black X's represent live and dead/dying fish, respectively (symbol area proportional to sample size for daily prevalences). Mean curves and 95% confidence regions from generalised additive models (blue: live, red: dead/dying). Left-hand grey region shows hatchery residence, grey regions on x-axis indicate period of transfer to another site, and vertical dotted lines show January 1st. Horizontal grey line indicates limit of detection. Note different scales.

A) prevalence B) intensity

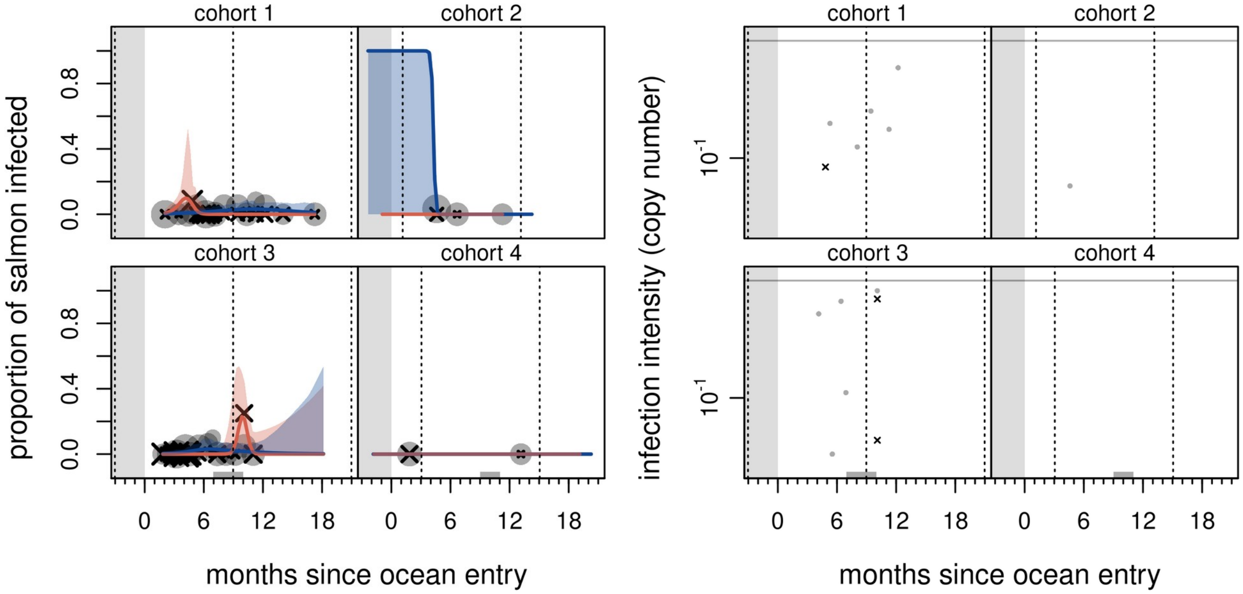

Figure S10. *Nucleospora salmonis* (Nu_sal) prevalence (A) and intensity (B) in farmed Atlantic salmon throughout four production cycles. Grey circles and black X's represent live and dead/dying fish, respectively (symbol area proportional to sample size for daily prevalences). Mean curves and 95% confidence regions from generalised additive models (blue: live, red: dead/dying). Left-hand grey region shows hatchery residence, grey regions on x-axis indicate period of transfer to another site, and vertical dotted lines show January 1st. Horizontal grey line indicates limit of detection. Note different scales.

A) prevalence B) intensity

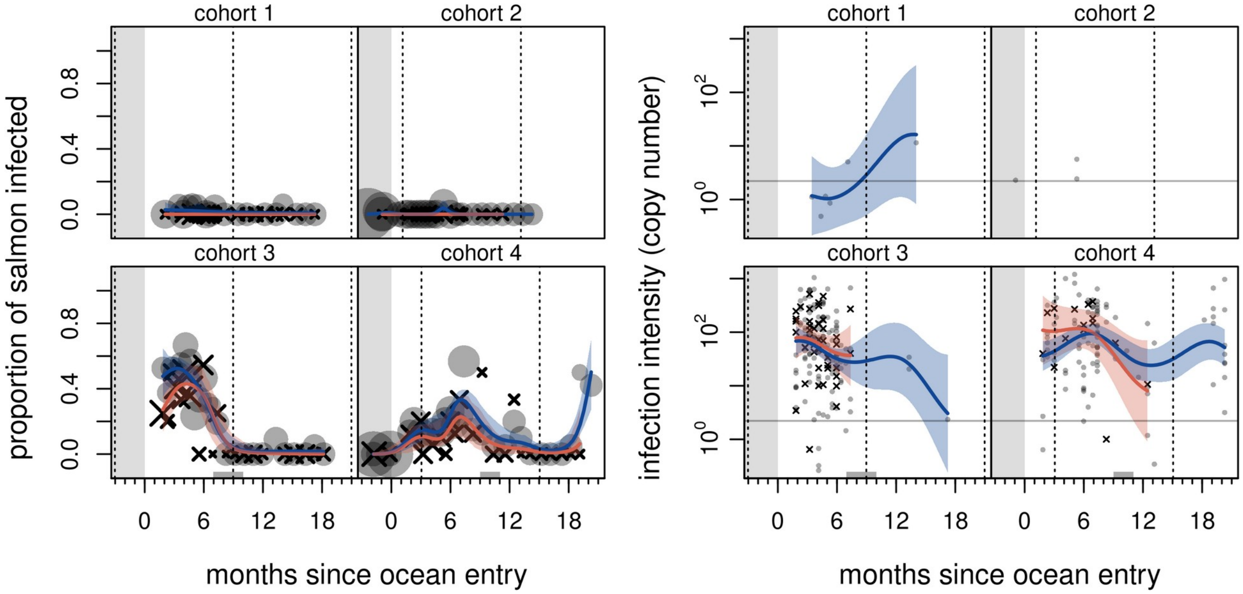

Figure S11. *Parvicapsula kabatai* (Pa_kab) prevalence (A) and intensity (B) in farmed Atlantic salmon throughout four production cycles. Grey circles and black X's represent live and dead/dying fish, respectively (symbol area proportional to sample size for daily prevalences). Mean curves and 95% confidence regions from generalised additive models (blue: live, red: dead/dying). Left-hand grey region shows hatchery residence, grey regions on x-axis indicate period of transfer to another site, and vertical dotted lines show January 1st. Horizontal grey line indicates limit of detection. Note different scales.

A) prevalence B) intensity

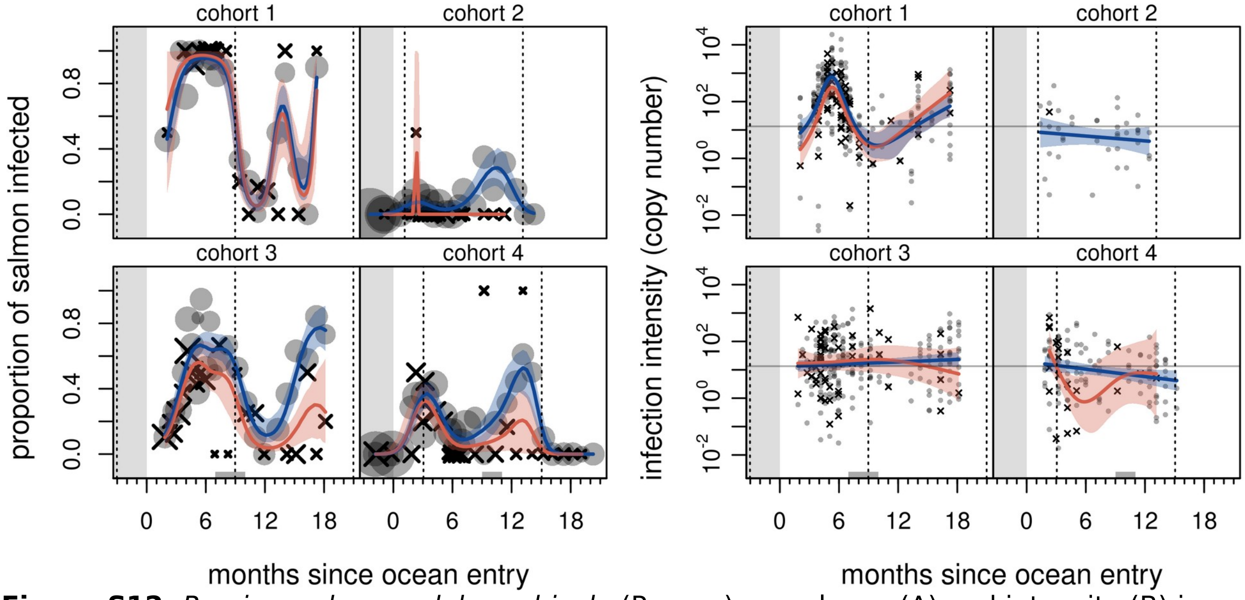

Figure S12. *Parvicapsula pseudobranchicola* (Pa_pse) prevalence (A) and intensity (B) in farmed Atlantic salmon throughout four production cycles. Grey circles and black X's represent live and dead/dying fish, respectively (symbol area proportional to sample size for daily prevalences). Mean curves and 95% confidence regions from generalised additive models (blue: live, red: dead/dying). Left-hand grey region shows hatchery residence, grey regions on x-axis indicate period of transfer to another site, and vertical dotted lines show January 1st. Horizontal grey line indicates limit of detection. Note different scales.

A) prevalence B) intensity

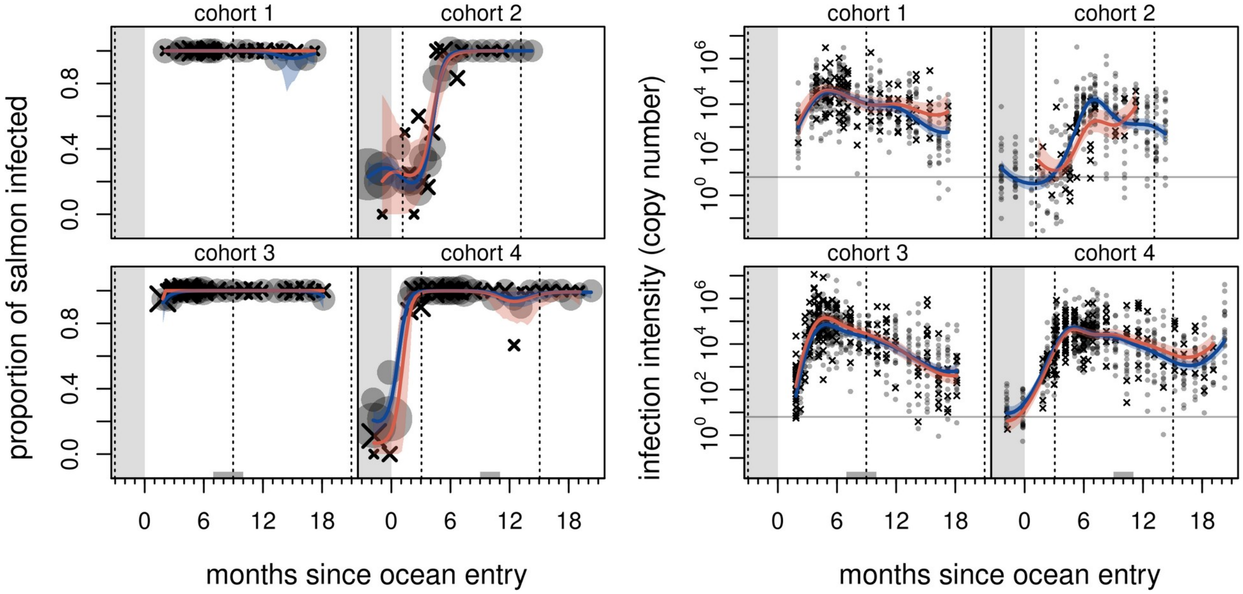

Figure S13. *Paranucleospora theridion* (Pa_ther) prevalence (A) and intensity (B) in farmed Atlantic salmon throughout four production cycles. Grey circles and black X's represent live and dead/dying fish, respectively (symbol area proportional to sample size for daily prevalences). Mean curves and 95% confidence regions from generalised additive models (blue: live, red: dead/dying). Left-hand grey region shows hatchery residence, grey regions on x-axis indicate period of transfer to another site, and vertical dotted lines show January 1st. Horizontal grey line indicates limit of detection. Note different scales.

A) prevalence B) intensity

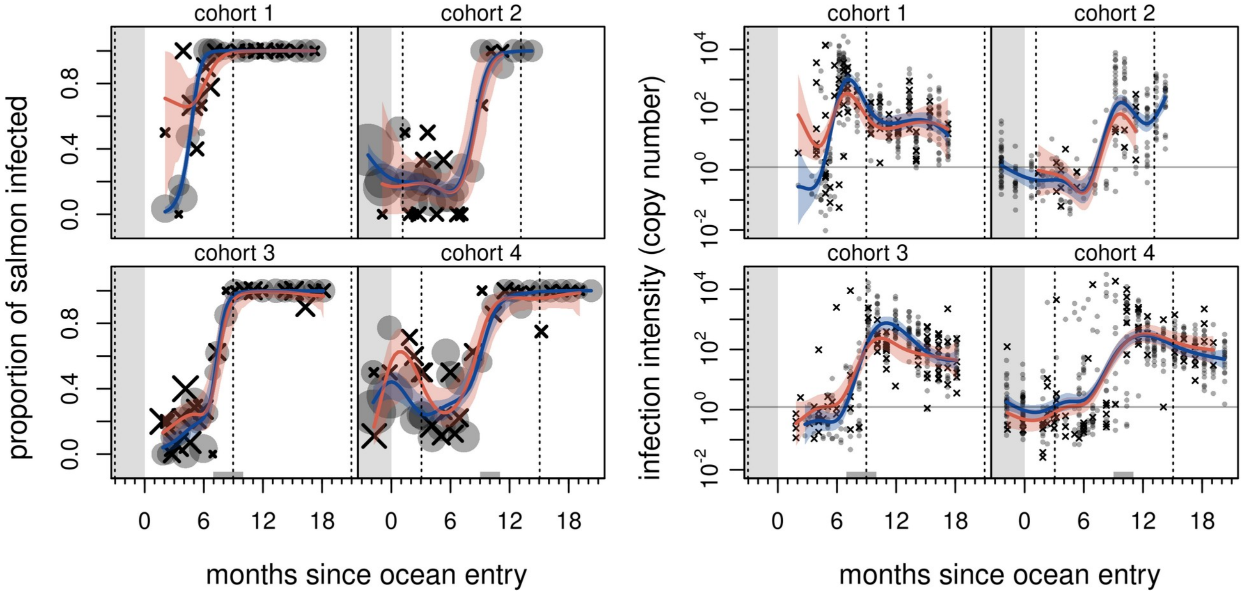

Figure S14. Piscine orthoreovirus (PRV) prevalence (A) and intensity (B) in farmed Atlantic salmon throughout four production cycles. Grey circles and black X's represent live and dead/dying fish, respectively (symbol area proportional to sample size for daily prevalences). Mean curves and 95% confidence regions from generalised additive models (blue: live, red: dead/dying). Left-hand grey region shows hatchery residence, grey regions on x-axis indicate period of transfer to another site, and vertical dotted lines show January 1st. Horizontal grey line indicates limit of detection. Note different scales.

A) prevalence B) intensity

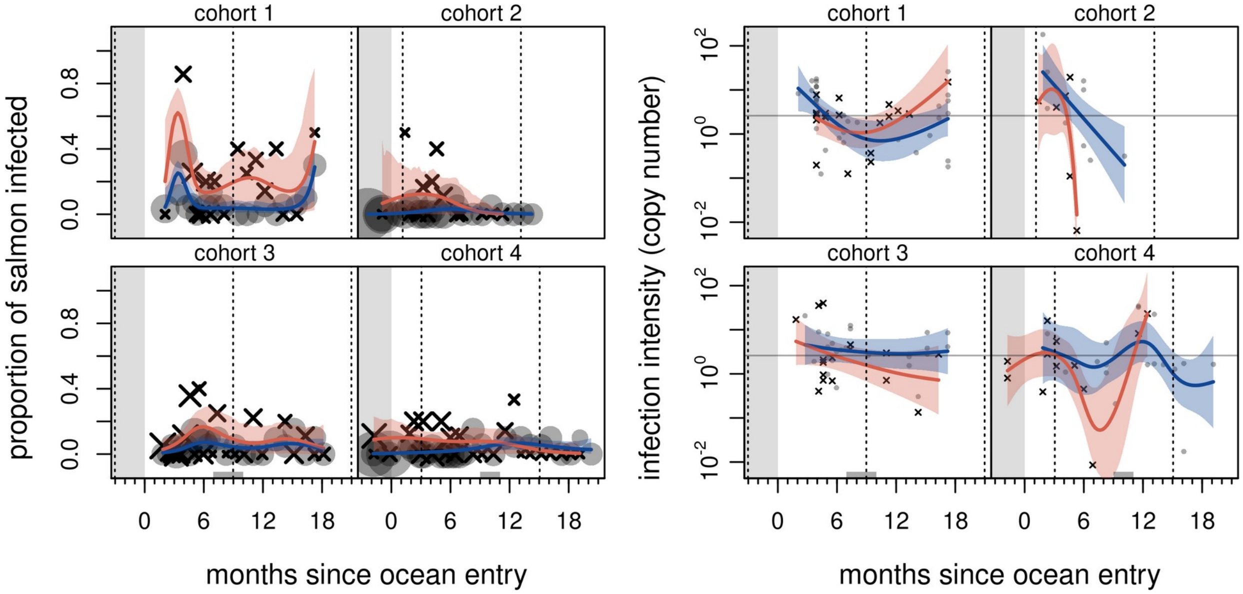

Figure S15. *Candidatus* Syngnamydia salmonis (C_S_sal) prevalence (A) and intensity (B) in farmed Atlantic salmon throughout four production cycles. Grey circles and black X's represent live and dead/dying fish, respectively (symbol area proportional to sample size for daily prevalences). Mean curves and 95% confidence regions from generalised additive models (blue: live, red: dead/dying). Left-hand grey region shows hatchery residence, grey regions on x-axis indicate period of transfer to another site, and vertical dotted lines show January 1st. Horizontal grey line indicates limit of detection. Note different scales.

A) prevalence B) intensity

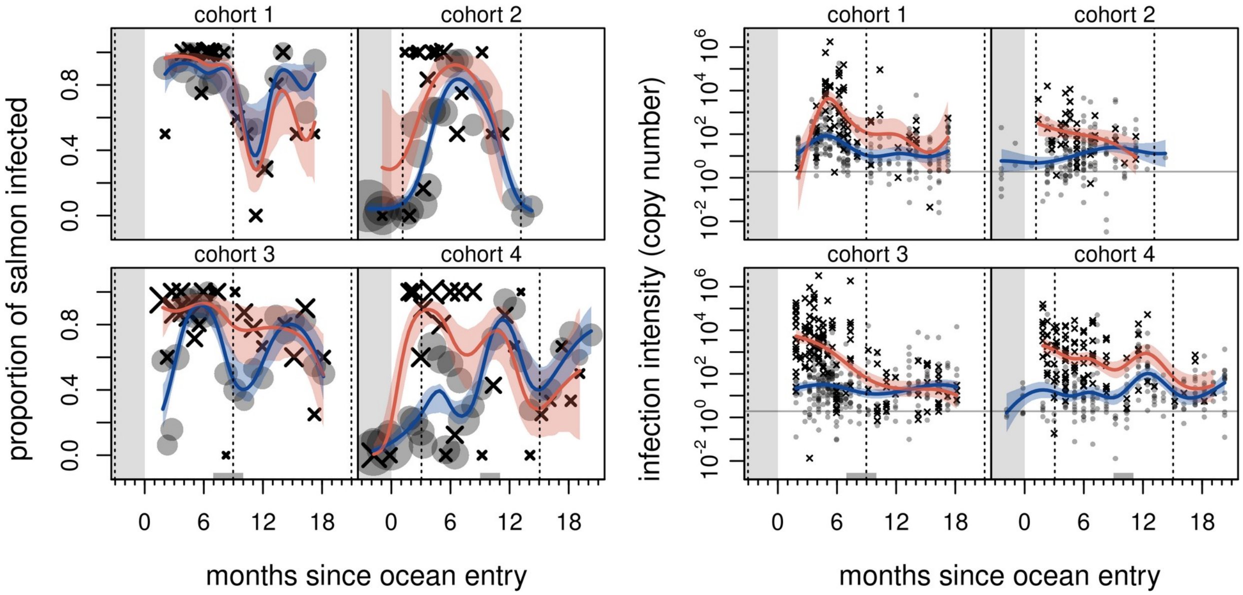

Figure S16. *Tenacibaculum maritimum* (Te_mar) prevalence (A) and intensity (B) in farmed Atlantic salmon throughout four production cycles. Grey circles and black X's represent live and dead/dying fish, respectively (symbol area proportional to sample size for daily prevalences). Mean curves and 95% confidence regions from generalised additive models (blue: live, red: dead/dying). Left-hand grey region shows hatchery residence, grey regions on x-axis indicate period of transfer to another site, and vertical dotted lines show January 1st. Horizontal grey line indicates limit of detection. Note different scales.

A) prevalence B) intensity

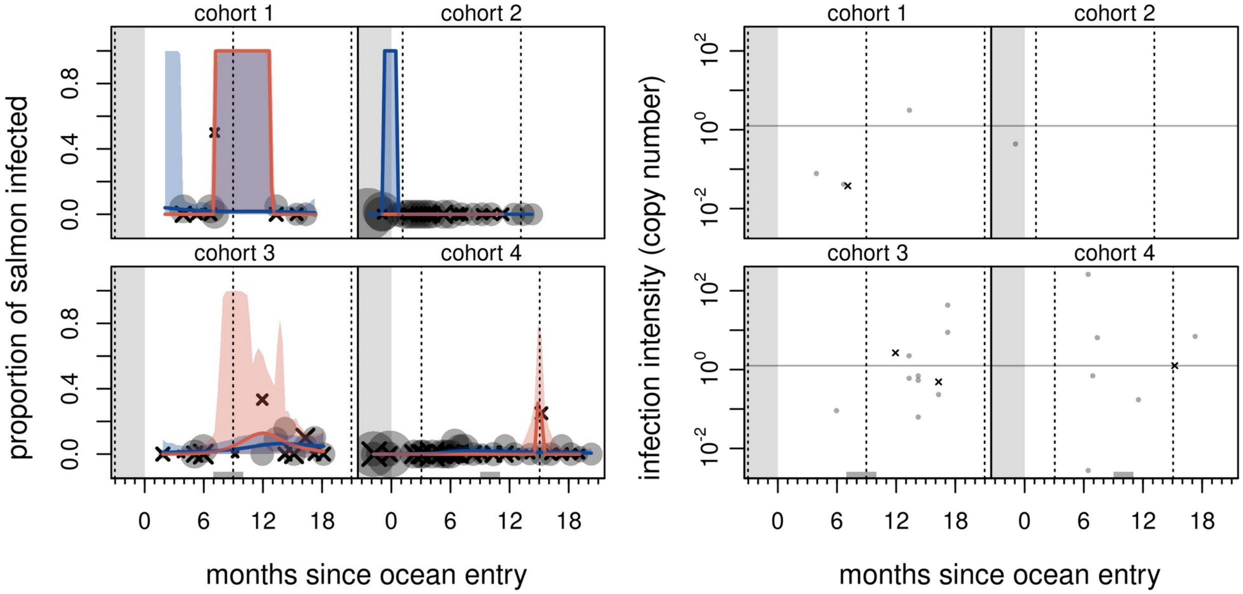

Figure S17. Putative totivirus (TOTIV) prevalence (A) and intensity (B) in farmed Atlantic salmon throughout four production cycles. Grey circles and black X's represent live and dead/dying fish, respectively (symbol area proportional to sample size for daily prevalences). Mean curves and 95% confidence regions from generalised additive models (blue: live, red: dead/dying). Left-hand grey region shows hatchery residence, grey regions on x-axis indicate period of transfer to another site, and vertical dotted lines show January 1st. Horizontal grey line indicates limit of detection. Note different scales.

A) prevalence B) intensity

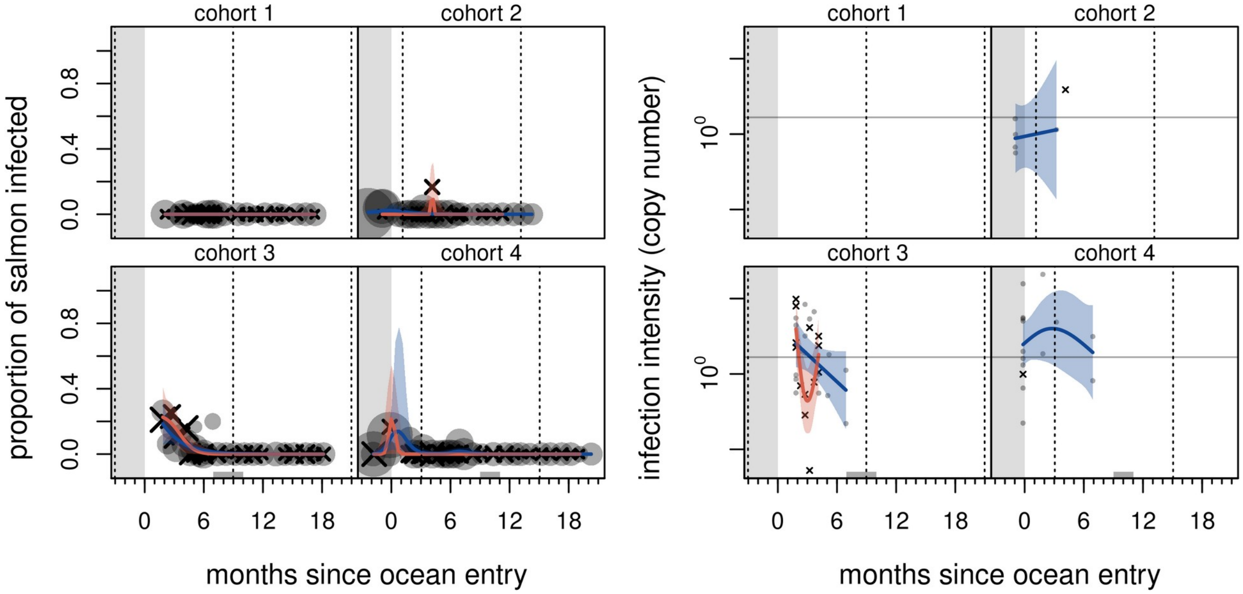

Figure S18. *Vibrio anguillarum* (Vi_ang) prevalence (A) and intensity (B) in farmed Atlantic salmon throughout four production cycles. Grey circles and black X's represent live and dead/dying fish, respectively (symbol area proportional to sample size for daily prevalences). Mean curves and 95% confidence regions from generalised additive models (blue: live, red: dead/dying). Left-hand grey region shows hatchery residence, grey regions on x-axis indicate period of transfer to another site, and vertical dotted lines show January 1st. Horizontal grey line indicates limit of detection. Note different scales.

A) prevalence B) intensity

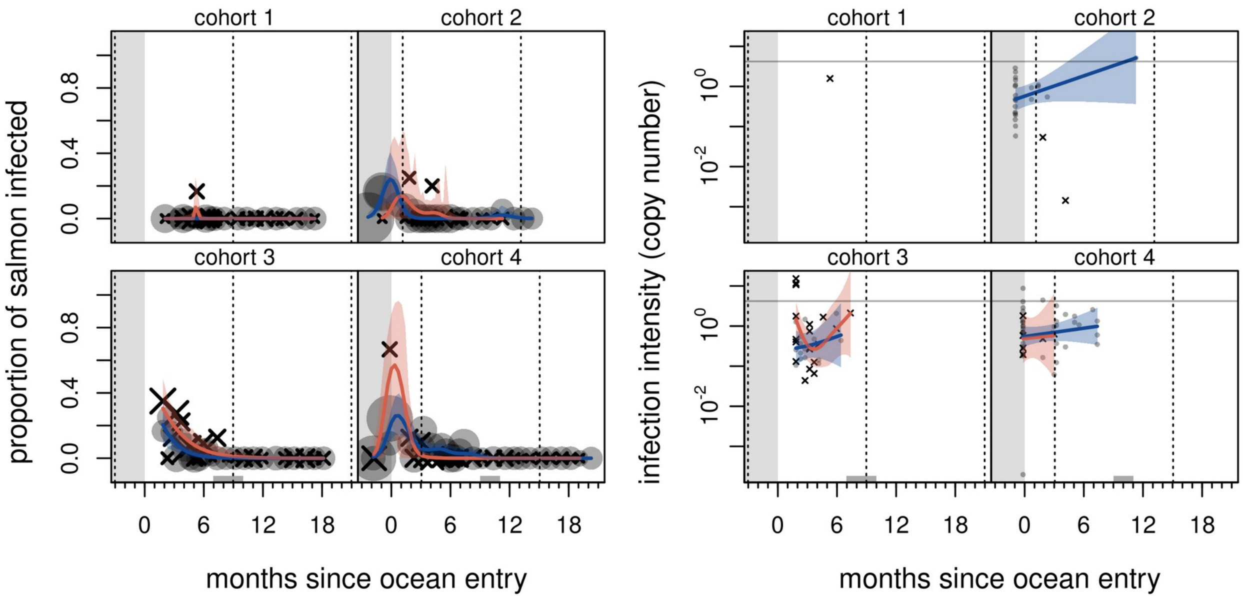

Figure S19. *Vibrio salmonicida* (Vi_sal) prevalence (A) and intensity (B) in farmed Atlantic salmon throughout four production cycles. Grey circles and black X's represent live and dead/dying fish, respectively (symbol area proportional to sample size for daily prevalences). Mean curves and 95% confidence regions from generalised additive models (blue: live, red: dead/dying). Left-hand grey region shows hatchery residence, grey regions on x-axis indicate period of transfer to another site, and vertical dotted lines show January 1st. Horizontal grey line indicates limit of detection. Note different scales.

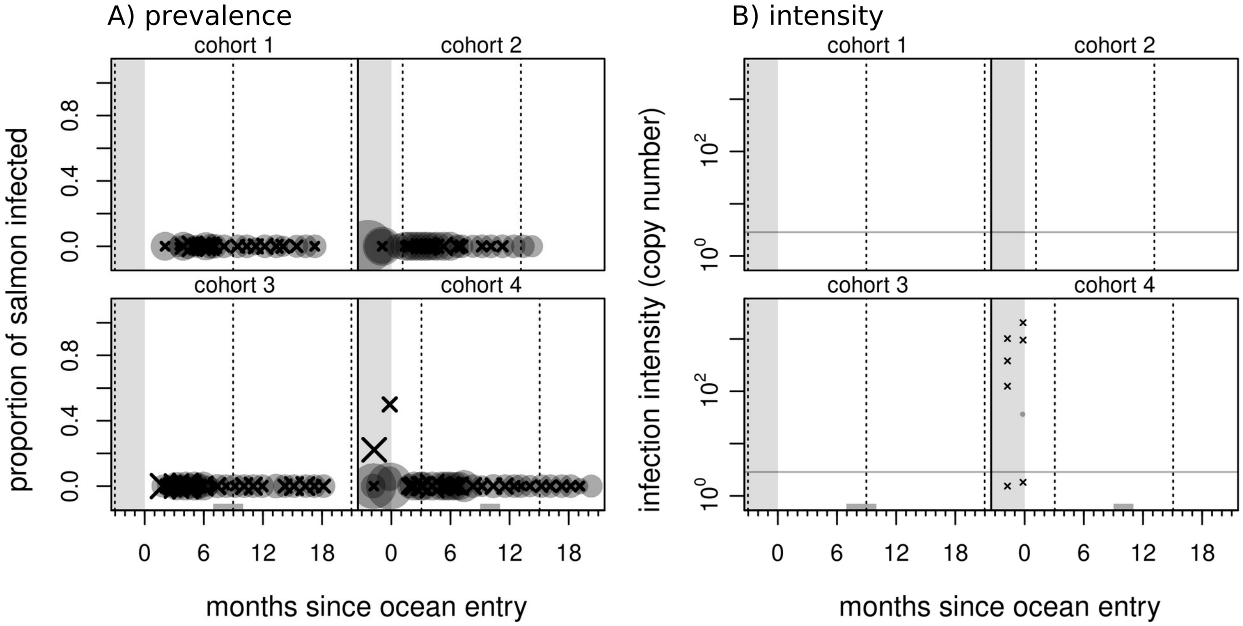

Figure S20. *Yersinia ruckeri* (Ye_ruc) prevalence (A) and intensity (B) in farmed Atlantic salmon throughout four production cycles. Grey circles and black X's represent live and dead/dying fish, respectively (symbol area proportional to sample size for daily prevalences). Left-hand grey region shows hatchery residence, grey regions on x-axis indicate period of transfer to another site, and vertical dotted lines show January 1st. Horizontal grey line indicates limit of detection. Note different scales.

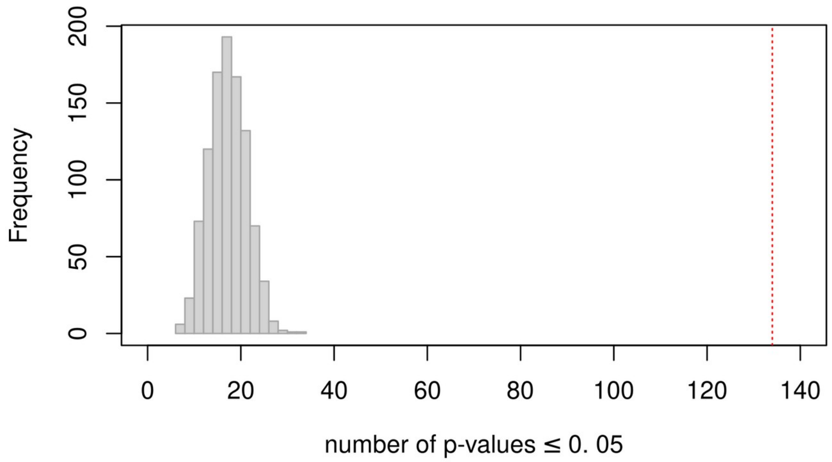

Figure S21. Number of p-values less than 0.05 for Spearman rank correlations between infectious-agent intensities in farmed Atlantic salmon in BC, Canada throughout four production cycles (Figure 5, main text). Histogram shows distribution from 1000 random iterations using data resampled with replacement; dotted red line shows observed value.

Supplemental references

Miller, K. M., I. A. Gardner, R. Vanderstichel, T. Burnley, A. D. Schulze, S. Li, A. Tabata, et al. 2016. *Report on the Performance Evaluation of the Fluidigm BioMark Platform for High Throughput Microbe Monitoring in Salmon*. Fisheries and Oceans Canada Canadian Science Advisory Secretariat.
